# New Insight into the History of Domesticated Apple: Secondary Contribution of the European Wild Apple to the Genome of Cultivated Varieties

**DOI:** 10.1371/journal.pgen.1002703

**Published:** 2012-05-10

**Authors:** Amandine Cornille, Pierre Gladieux, Marinus J. M. Smulders, Isabel Roldán-Ruiz, François Laurens, Bruno Le Cam, Anush Nersesyan, Joanne Clavel, Marina Olonova, Laurence Feugey, Ivan Gabrielyan, Xiu-Guo Zhang, Maud I. Tenaillon, Tatiana Giraud

**Affiliations:** 1 CNRS, Laboratoire Ecologie Systématique et Evolution – UMR8079, Orsay, France; 2 Université Paris Sud, Orsay, France; 3 AgroParisTech, Orsay, France; 4 Plant Research International, Wageningen UR Plant Breeding, Wageningen, The Netherlands; 5 Growth and Development Group, Plant Sciences Unit, Institute for Agricultural and Fisheries Research (ILVO), Melle, Belgium; 6 INRA, IRHS, PRES UNAM, SFR QUASAV, Beaucouzé, France; 7 Université d'Angers, IRHS, PRES UNAM, SFR QUASAV, Angers, France; 8 Agrocampus Ouest, IRHS, PRES UNAM, SFR QUASAV, Angers, France; 9 Institute of Botany, Department of Plant Taxonomy, Armenian National Academy of Sciences, Yerevan, Armenia; 10 Biological Institution, Tomsk State University, Tomsk, Russia; 11 Department of Plant Pathology, Shandong Agricultural University, Taian, China; 12 CNRS, UMR de Génétique Végétale, INRA/CNRS/Univ Paris-Sud, Gif-sur-Yvette, France; University of Georgia, United States of America

## Abstract

The apple is the most common and culturally important fruit crop of temperate areas. The elucidation of its origin and domestication history is therefore of great interest. The wild Central Asian species *Malus sieversii* has previously been identified as the main contributor to the genome of the cultivated apple (*Malus domestica*), on the basis of morphological, molecular, and historical evidence. The possible contribution of other wild species present along the Silk Route running from Asia to Western Europe remains a matter of debate, particularly with respect to the contribution of the European wild apple. We used microsatellite markers and an unprecedented large sampling of five *Malus* species throughout Eurasia (839 accessions from China to Spain) to show that multiple species have contributed to the genetic makeup of domesticated apples. The wild European crabapple *M. sylvestris*, in particular, was a major secondary contributor. Bidirectional gene flow between the domesticated apple and the European crabapple resulted in the current *M. domestica* being genetically more closely related to this species than to its Central Asian progenitor, *M. sieversii*. We found no evidence of a domestication bottleneck or clonal population structure in apples, despite the use of vegetative propagation by grafting. We show that the evolution of domesticated apples occurred over a long time period and involved more than one wild species. Our results support the view that self-incompatibility, a long lifespan, and cultural practices such as selection from open-pollinated seeds have facilitated introgression from wild relatives and the maintenance of genetic variation during domestication. This combination of processes may account for the diversification of several long-lived perennial crops, yielding domestication patterns different from those observed for annual species.

## Author Summary

The apple, one of the most ubiquitous and culturally important temperate fruit crops, provides us with a unique opportunity to study the process of domestication in trees. The number and identity of the progenitors of the domesticated apple and the erosion of genetic diversity associated with the domestication process remain debated. The Central Asian wild apple has been identified as the main progenitor, but other closely related species along the Silk Route running from Asia to Western Europe may have contributed to the genome of the domesticated crop. Using rapidly evolving genetic markers to make inferences about the recent evolutionary history of the domesticated apple, we found that the European crabapple has made an unexpectedly large contribution to the genome of the domesticated apple. Bidirectional gene flow between the domesticated apple and the European crabapple resulted in the domesticated apple being currently more similar genetically to this secondary genepool than to the ancestral progenitor, the Central Asian wild apple. We found that domesticated apples have evolved over long time scales, with contributions from at least two wild species in different geographic areas, with no significant erosion of genetic diversity. This process of domestication and diversification may be common to other fruit trees and contrasts with the models documented for annual crops.

## Introduction

Domestication is a process of increasing codependence between plants and animals on the one hand, and human societies on the other [1], [2]. The key questions relating to the evolutionary processes underlying domestication concern the identity and geographic origin of the wild progenitors of domesticated species [3], the nature of the genetic changes underlying domestication [4], [5], the tempo and mode of domestication (*e.g.*, rapid transition *versus* protracted domestication) [6] and the consequences of domestication for the genetic diversity of the domesticated species [7], [8], [9], [10]. An understanding of the domestication process provides insight into the general mechanisms of adaptation and the history of human civilization, but can also guide modern breeding programs aiming to improve crops or livestock species further [11], [12].

Plant domestication has mostly been studied in seed-propagated annual crops, in which strong domestication bottlenecks have often been inferred, especially in selfing annuals, such as foxtail millet, wheat and barley [11], [13], [14], [15], [16], [17]. Genetic data have suggested that domestication or the spread of domesticated traits has been fairly rapid in some annual species (*e.g*, maize or sunflower), with limited numbers of populations or species contributing to current diversity [10], [18], [19], [20], [21], [22]. In contrast, a combination of genetics and archaeology suggested a protracted model of domestication for other annual crops, and in particular for the origin of wheat or barley in the Fertile Crescent [11], [23]. However, the genetic consequences of domestication have been little investigated in long-lived perennials, such as fruit trees [24], [25], [26]. Trees have several biological features that make them fascinating and original models for investigating domestication: they are outcrossers with a long lifespan and a long juvenile phase, and tree populations are often large and connected by high levels of gene flow [27], [28].

Differences in life-history traits probably result in marked differences in the mode and speed of evolution between trees and seed-propagated selfing annuals [27], [28], [29]. For example, outcrossing may tend to make domestication more difficult, in part because the probability of fixing selected alleles is lower than in selfing crops [6], [13]. The combination of self-incompatibility and a long juvenile phase also results in highly variable progenies, making breeding a slow and expensive process, and rendering crop improvement difficult. The development of vegetative propagation based on cuttings or grafting has been a key element in the domestication of long-lived perennials, allowing the maintenance and spread of superior individuals despite self-incompatibility [30]. However, the use of such techniques has further decreased the number of sexual cycles in tree crops since the initial domestication event, adding to the effect of long juvenile phases in limiting the genetic divergence between cultivated trees and their wild progenitors [30], [31], [32], [33]. Thus, domestication can generally be considered more recent, at least in terms of the number of generations, in fruit tree crops than in seed-propagated selfing annuals.

Given the slow process of selection and the limited number of generations in which humans could exert selection, the protracted nature of the domestication process in trees has probably resulted in limited bottlenecks [25], [31] and in a weaker domestication syndrome [34] than in seed-propagated annuals. Nevertheless, many cultivated fruit trees clearly display morphological, phenotypic and physiological features typical of a domestication syndrome, such as large fruits and high sugar or oil content [32], [35]. Many aspects of fruit tree domestication have been little studied [25]. Consequently, most of the hypotheses concerning the consequences of particular features of trees for their domestication/diversification remain to be tested. Recent studies on grapevines, almond and olive trees have provided illuminating insights, such as the importance of outcrossing and interspecific hybridization [36], [37], [38], but additional studies of other species are required to draw more general conclusions.

Here, we investigated the origins of the domesticated apple *Malus domestica* Borkh., one of the most emblematic and widespread fruit crops in temperate regions [35]. A form of apple corresponding to extant domestic apples appeared in the Near East around 4,000 years ago [39], at a time corresponding to the first recorded uses of grafting. The domesticated apple was then introduced into Europe and North Africa by the Greeks and Romans and subsequently spread worldwide [35]. While the ancestral progenitor has been clearly identified as being *M. sieversii*, the identity and relative contributions of other wild species present along the Silk route that have contributed to the genetic makeup of apple cultivars remain largely unknown. This is surprising given the potential importance of this knowledge for plant breeding and for our understanding of the process of domestication in fruit trees.

The wild Central Asian species *M. sieversii* (Ldb.) M. Roem has been identified as the main contributor to the *M. domestica* genepool based on similarities in fruit and tree morphology, and genetic data [40], [41], [42], [43]. The Tian Shan forests were identified as the geographic area in which the apple was first domesticated, on the basis of the considerable intraspecific morphological variability of wild apple populations in this region [44], [45]. Nucleotide variation for 23 DNA fragments even suggested that *M. sieversii* and *M. domestica* belonged to a single genepool (which would be called *M. pumila* Mill.), with phylogenetic networks showing an intermingling of individuals from the two taxa [43]. Some authors have also suggested possible contributions of additional wild species present along the Silk Route: *M. baccata* (L.) Borkh, which is native to Siberia, *M. orientalis* Uglitz., a Caucasian species present along western sections of the ancient trade routes, and *M. sylvestris* Mill. (European crabapple), a species native to Europe [46], [47], [48], [49]. These hypotheses were based on the history of human migration and trade, the lack of phylogenetic resolution between *M. domestica* and these four wild species [41], [42], genetic evidence of hybridization at a local scale between domesticated apple and *M. sylvestris* [40], and the recent finding of sequence haplotype sharing between *M. sylvestris* and *M. domestica* [50]. However, such secondary contributions remain a matter of debate, mostly due to the difficulty of distinguishing introgression from incomplete lineage sorting [43], [50], [51]. The three wild species occurring along the Silk Route all bear small, astringent, tart fruits. None of these species has the fruit quality of *M. sieversii*, but they may have contributed other valuable horticultural traits, such as later flowering, resistance to pests and diseases, capacity for longer storage or climate adaptation. The organoleptic properties of the fruits of these wild species may also have been selected during domestication, for the preparation of apple-based beverages, such as ciders [46], [52]. Cider apples are indeed smaller, bitter and more astringent than dessert apples and bear some similarity to *M. sylvestris* apples. There is also evidence to suggest that Neolithic and Bronze Age Europeans were already making use of *M. sylvestris* [39].

**Figure 1:**
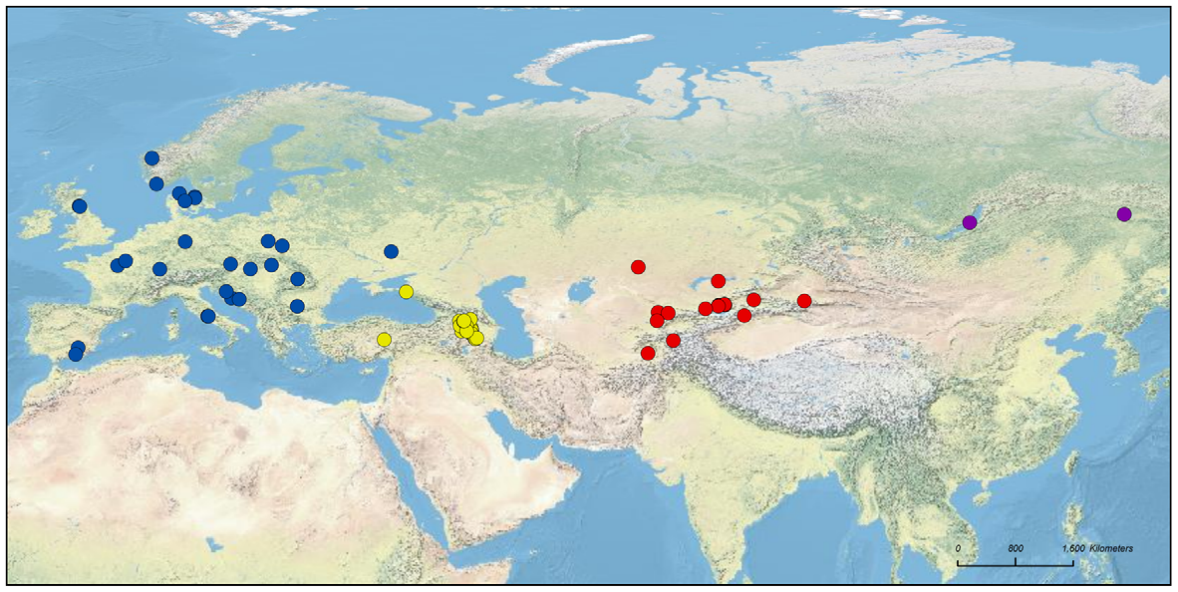
Geographic origins of the samples of the four wild *Malus* species used: *M. sylvestris* (blue), *M. orientalis* (yellow), *M. baccata* (purple), and *M. sieversii* (red). Samples of unknown origin (*N* = 28) were not projected onto the map.

In this study, we used a comprehensive set of apple accessions sampled across Eurasia (839 accessions from China to Spain; Figure 1 and Figure S1; Table S1) and 26 microsatellite markers distributed evenly across the genome to investigate the following questions: 1) Is there evidence for population subdivision within and between the five taxa *M. domestica*, *M. baccata*, *M. orientalis*, *M. sieversii* and *M. sylvestris*? 2) How large is the contribution of wild species other than the main progenitor, *M. sieversii*, to the genome of *M. domestica*? 3) Does *M. domestica* have a genetic structure associated with its different possible uses (*i.e.*, differences between cider and dessert apples)? 4) What consequences have domestication, subsequent crop improvement and vegetative propagation by grafting had for genetic variation in cultivated apples? Most of our samples of *M. domestica* corresponded to cultivars from Western Europe (Figure 1 and Figure S1), as almost all the cultivars available in modern collections (including American, Australasian cultivars) are of European ancestry and this region is therefore the most relevant area for the detection of possible secondary introgression from the European crabapple.

## Results

### High diversity and low deviations from random mating expectations within species

Our sampling scheme (Figure 1 and Figure S1), based on the collection of a single tree for each apple variety, was designed to avoid the sampling of clones. However, there may still be some clonality if some varieties differing by only a few mutations were propagated by grafting. We corrected for this potential clonality, using the clonal assignment procedures implemented in GENODIVE [53]. We found no pair of samples assigned to the same clonal lineage unless using a threshold of 22 pairwise differences between multilocus genotypes, indicating that our samples did not include any clonal genotypes (the threshold corresponds to the maximum genetic distance allowed between genotypes deemed to belong to the same clonal lineage).

Many apple cultivars, including modern cultivars in particular, share recent common ancestors, and siblings or clones of wild species can also be collected unintentionally in the field. Because these features could result in a spurious genetic structure due to the presence of closely related individuals in the dataset, we checked for the presence of groups of related individuals in our dataset between *M. domestica* cultivars and between the individuals of each wild species. The percentage of pairs with a pairwise relatedness (*r_xy_*) greater than 0.5 (*i.e.*, full sibs) was: 0.4% in *M. domestica* (*N* = 168 pairs), 0.3% in *M. sieversii* (*N* = 79), 0.004% in *M. orientalis* (*N* = 20), and 0.7% in *M. baccata* (*N* = 40). For *M. sylvestris*, no individual pair with *r_xy_*>0.5 was identified. However, the distribution of pairwise relatedness *r_xy_* among *M. domestica* cultivars did not deviate significantly from a Gaussian distribution centred on 0 and with a low variance (Fisher's exact test, *P*≈1, standard deviation = 0.11, Figure S2). This suggests that closely related cultivars are unlikely to have biased subsequent analyses of population structure. We also checked for the limited effect of relatedness on our conclusions by performing all analyses of population subdivision on both the full dataset and a pruned dataset excluding related individuals (see below).

We tested the null hypothesis of random mating within each species by calculating *F_IS_*, which measures inbreeding. All five *Malus* species had relatively low values of *F_IS_*, although all were significantly different from zero (Table 1), suggesting that each species corresponded to an almost random mating unit. This is consistent with the self-incompatibility system of these species and indicates a lack of widespread groups of related individuals in *M. domestica*. Low *F_IS_* values at species level also indicate a lack of population structure within species. The higher values of *F_IS_* observed in *M. baccata* probably resulted from the occurrence of null alleles, as the microsatellite markers were developed in *M. domestica*, to which *M. baccata* is the most distantly related (Table 2). The lowest *F_IS_* value was that obtained for *M. domestica*, reflecting outcrossing between dissimilar parents in breeding programs, or that selection targeted higher levels of heterozygosity [54].

**Table 1:** Summary of genetic variation in the five *Malus* species.

	*H_O_*	*H_E_*	*F_IS_*	*A_r_*	*A_p_*	A_p_*	*P_NA_*
*M. domestica*	0.81	0.83	0.02***	8.0	0.8	1.2	0.02
*M. sieversii*	0.77	0.82	0.07***	8.0	1.1	1.2	0.03
*M. sylvestris*	0.75	0.87	0.14***	9.9	1.7	2.5	0.02
*M. orientalis*	0.79	0.84	0.06***	8.8	2.1	1.9	0.03
*M. baccata*	0.56	0.75	0.24***	7.8	1.4	2.1	0.12

*H_O_* and *H_E_*: observed and expected heterozygosity, respectively, *F_IS_*: inbreeding coefficient, *A_r_* and *A_p_*: allelic richness and private allele richness averaged across loci, respectively, estimated by rarefaction using a standardized sample size of 22, A_p_*: private allele richness averaged across loci using the pruned dataset without hybrids in both wild and cultivated species, estimated by rarefaction using a standardized sample size of 12; *P_NA_*: proportion of null alleles,

***: : *P*-value<0.0001.

**Table 2:** Pairwise differentiation (*F_ST_*) between the five *Malus* species.

	*M. baccata*	*M. sylvestris*	*M. domestica*	*M. sieversii*
*M. sylvestris*	0.1683	-	-	-
*M. domestica*	0.1505	0.0056	-	-
*M. sieversii*	0.1457	0.0818	0.0639	-
*M. orientalis*	0.1337	0.0579	0.0494	0.0393

All *F_ST_* values were significant (*P*<0.001).

### The five *Malus* species form well separated genetic clusters

**Figure 2:**
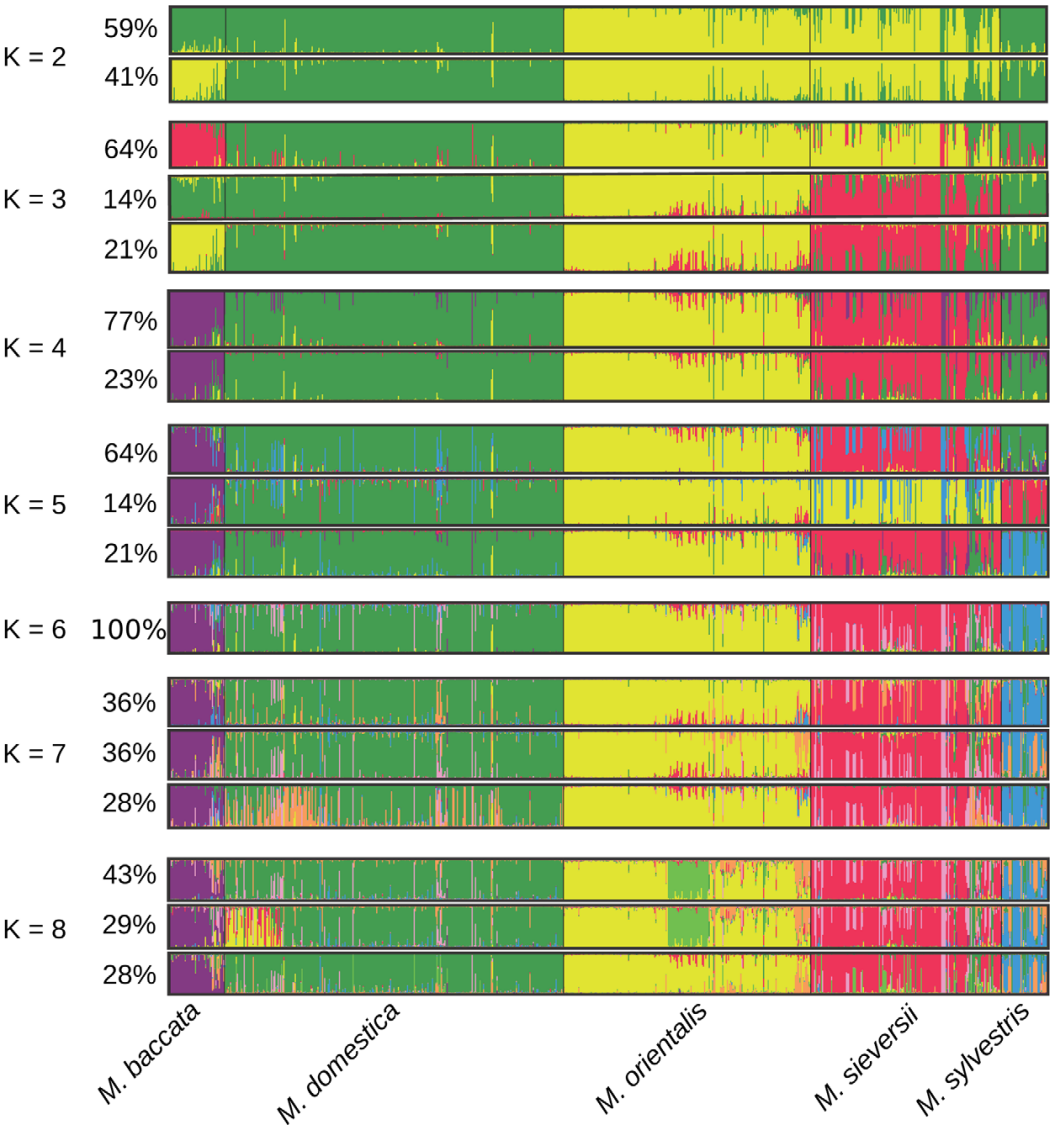
Proportions of ancestry of *Malus* genotypes from five species (*N* = 770) from *K* = 2 to *K* = 8 ancestral genepools (“clusters”) inferred with the STRUCTURE program. Each individual is represented by a vertical bar, partitioned into *K* segments representing the amount of ancestry of its genome in *K* clusters. When several clustering solutions (“modes”) were represented within replicate runs, the proportion of simulations represented by each mode is given.

We used the ‘admixture model’ implemented in STRUCTURE 2.3 [55] to infer population structure and introgression. Analyses were run for population structure models assuming *K* = 1 to *K* = 8 distinct clusters (Figure 2). The *ΔK* statistic, designed to identify the most relevant number of clusters by determining the number of clusters beyond which there is no further increase in likelihood [56], was greatest for *K* = 3 (*ΔK* = 6249, *Pr|ln L* = −78590). However, the clusters identified at higher *K* values may also reveal a genuine and biologically relevant genetic structure, provided that they are well delimited [57]. The five *Malus* species were clearly assigned to different clusters for models assuming *K*≥6 clusters and for a minor clustering solution (“mode”) at *K* = 5 (Figure 2). The major mode (*i.e.*, the clustering solution found in more than 60% of the simulation replicates) observed at *K* = 5 grouped together *M. sylvestris* and *M. domestica* genotypes. Increasing the number of clusters above *K* = 6 identified no additional well-delimited clusters corresponding to a subdivision of a previous cluster. Instead, it simply introduced heterogeneity into membership coefficients, indicating that the clustering of the five *Malus* species into separate genepools was the most relevant clustering solution. We checked that the presence of related pairs of cultivars in our dataset did not bias clustering results, by repeating the analysis on a pruned dataset (*N* = 489) excluding all related individuals in wild and cultivated species (*i.e.*, excluding all pairs with *r_xy_*≥0.5). Similar results were obtained, with the same five distinct clusters identified as for the full dataset.

We estimated the genetic differentiation between the five *Malus* species by calculating pairwise *F_ST_* (Table 2). All *F_ST_* values were highly significant (*P*<0.001) and seemed to indicate a West to East differentiation gradient of *M. domestica* with the wild species. The highest level of differentiation was that between *M. baccata* and the other *Malus* species, and the lowest level of differentiation was that between *M. domestica* and the westernmost species, *M. sylvestris* (Table 2). *Malus domestica* was markedly more differentiated from its main progenitor *M. sieversii* (*F_ST_* = 0.0639) than from the European *M. sylvestris* (*F_ST_* = 0.006) and it was only slightly less differentiated from the Caucasian *M. orientalis* (*F_ST_* = 0.049).

### No bottleneck during apple domestication

We first searched for footprints of a domestication bottleneck by comparing levels of microsatellite variation in *M. domestica* and wild species. There was no significant difference in genetic diversity (as measured by expected heterozygosity, *H_E_*) between *M. domestica* and *M. baccata*, *M. orientalis* or *M. sieversii*, but *H_E_* was significantly higher in *M. sylvestris* than in *M. domestica* (Table 1). Significant differences in allelic richness (*A_r_*) were found between *M. domestica* and *M. orientalis* (Wilcoxon signed rank test, *P* = 0.03) or *M. sylvestris* (*P*<10^−8^), but not between *M. domestica* and either *M. baccata* (*P* = 0.9) or *M. sieversii* (*P* = 0.9) (Table 1).

We used the method implemented in the BOTTLENECK program [58], comparing the expected heterozygosity estimated from allele frequencies with that estimated from the number of alleles and the sample size, which should be identical for a neutral locus in a population at mutation-drift equilibrium. Inferences about historical changes in population size are based on the prediction that the expected heterozygosity estimated from allele frequencies decreases faster than that estimated under a given mutation model at mutation-drift equilibrium in populations that have experienced a recent reduction in size. BOTTLENECK analysis showed no significant deviation from mutation-drift equilibrium in any of the five species, under either stepwise or two-phase models of microsatellite evolution (one-tailed Wilcoxon signed rank test, *P*>0.95). We therefore detected no signal of a demographic bottleneck associated with the domestication of apples.

### Variable recent contributions of wild relative species to the *M. domestica* genepool, with the strongest introgression from *M. sylvestris*

We used the admixture coefficients estimated by STRUCTURE to assess the recent contribution of the various wild species to the *M. domestica* genepool. STRUCTURE analyses of the full dataset showed some admixture among *Malus* species for the minor mode separating the five species at *K* = 5. Admixture coefficients were higher between *M. domestica* and *M. sylvestris* (*α* = 0.23) than between *M. domestica* and respectively *M. sieversii* (*α* = 0.06), *M. orientalis* (*α* = 0.034) and *M. baccata* (*α* = 0.032).

**Figure 3:**
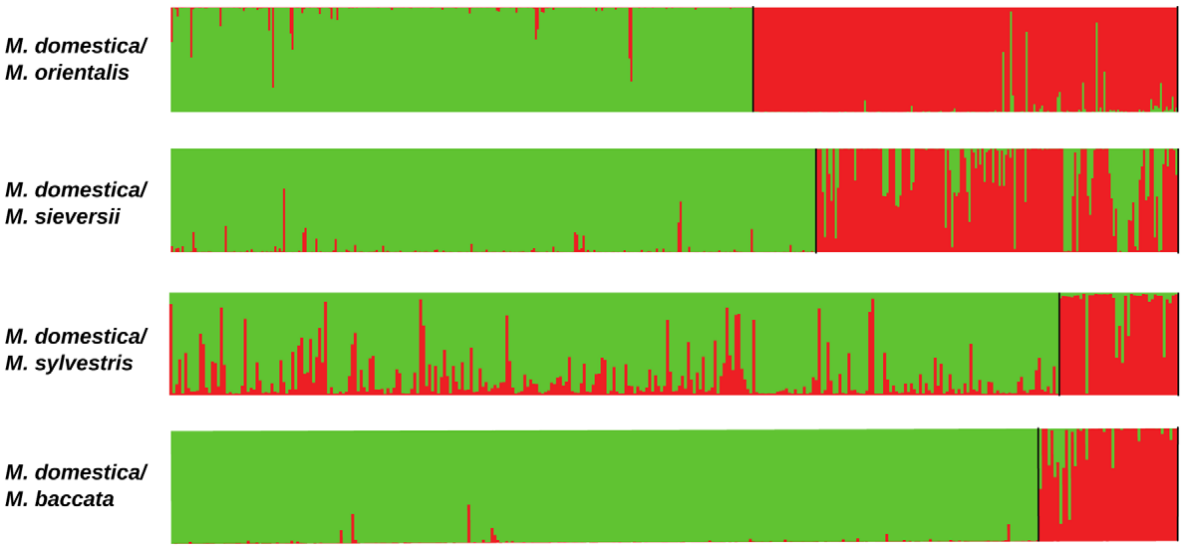
Proportions of ancestry in two ancestral genepools inferred with the STRUCTURE program, based on datasets including *M. domestica* (green, *N* = 299) and each of the four wild *Malus* species (red). The x-axis is not to scale (details in Table S2).

We further analysed the contribution of each wild species to the genome of *M. domestica* by running STRUCTURE separately on each pair of species including *M. domestica* (Figure 3; Table 3 and Table S2). *Malus domestica* genotypes with membership coefficients ≥0.20 in a wild species genepool were considered to display introgression. Using this somehow arbitrary cut-off value, STRUCTURE analyses revealed that 26% of *M. domestica* cultivars displayed introgression from the European crabapple, *M. sylvestris* (Table 3 and Table S2). By contrast, only 2%, 3% and 0.02% of the *M. domestica* genotypes displayed introgression from *M. sieversii*, *M. orientalis* and *M. baccata*, respectively (Table 3 and Table S2). The *M. domestica* cultivars displaying admixture with the *M. sylvestris* genepool were mostly Russian (*e.g.*, “Antonovka”, “Antonovka kamenicka”, “Novosibirski Sweet”, “Yellow transparent”), French (*e.g.*, “Blanche de St Anne”, “St Jean”, “Api” and “Michelin”) and English (*e.g.*, “Worcester Pearmain” and “Fiesta”). The M9 dwarf apple cultivar (“Paradis jaune de Metz”, [59]) commonly used as a rootstock also appeared to display introgression from the European crabapple (proportion of ancestry in the *M. domestica* genepool: 0.28; Table S2). When French cultivars were removed from the dataset (*N* = 89) and pairwise STRUCTURE analyses were repeated for all species pairs including *M. domestica*, 18% of cultivars displayed introgression from *M. sylvestris*, including commercial cultivars such as Granny Smith, Michelin, Antonovka and Ajmi (Figure S3) with a mean membership coefficient of *M. sylvestris* into *M. domestica* genepool of 47%. *Malus sylvestris* thus appears to have made a significant contribution to the *M. domestica* genepool through recent introgression, building on the more ancient contribution (see below) of the Asian wild species *M. sieversii*. We also note that a few *M. domestica* individuals appeared to display introgression from several wild species (Table S2), and that *M. baccata* ornamental cultivars, such as *M. baccata flexilis*, *M. baccata* Hansen's and *M. baccata gracilis*, were partially or even mostly assigned (from 32% to >80%) to the *M. domestica* genepool (Table S3).

**Table 3:** Mean proportions of assignment to each of the two species in species pair comparisons (K = 2) including *M. domestica* (Genepool 1) and each of the four wild *Malus* species (Genepool 2).

Species pairs	Genepool 1	Genepool 2
*M. domestica*	0.841	0.159
*M. sylvestris*	0.119	0.881
*M. domestica*	0.993	0.007
*M. baccata*	0.104	0.896
*M. domestica*	0.980	0.020
*M. orientalis*	0.030	0.970
*M. domestica*	0.981	0.019
*M. sieversii*	0.231	0.769

### Wild Central Asian apple origin of the *M. domestica* genepool

Previous studies [43], [50], [60] identified the Central Asian wild apple *M. sieversii* as the main progenitor of *M. domestica* on the basis of DNA sequences. Due to the large contribution by *M. sylvestris* detected in our dataset, corresponding mostly to Western European cultivars, *M. domestica* and *M. sylvestris* appeared to be the most closely related pair of species in our analyses of microsatellite markers. We investigated the more ancient contribution of *M. sieversii* to the *M. domestica* genepool, by reassessing the genetic differentiation between species in analyses restricted to “pure” individuals (*i.e.*, assigned at ≥0.9 to their respective genepools) from both wild and cultivated species. All *F_ST_* values were highly significant (*P*<0.001), but the ranking of *F_ST_* values between *M. domestica* and the various wild species was affected: the highest differentiation was still observed between *M. domestica* and *M. baccata* (*F_ST_* = 0.22), but the lowest differentiation was observed between *M. domestica* and *M. sieversii* (*F_ST_* = 0.11). Regarding the differentiation between *M. sylvestris* and *M. domestica*, we observed the opposite of what was found with the full dataset: *M. sylvestris* appeared to be more strongly differentiated (*F_ST_* = 0.14) from *M. domestica* than *M. sieversii*. Thus, by removing signals of recent introgression between cultivated and wild species we were able to confirm that *M. sieversii* was the initial progenitor of *M. domestica*.

### Recent introgression from *M. domestica* into wild species

The finding of a significant level of introgression from wild species into cultivated apple suggested that gene flow might also have occurred in the opposite direction. STRUCTURE analyses of pairs of species confirmed this hypothesis (Figure 3), revealing possible introgression of genetic material into *M. sylvestris*, *M. baccata*, *M. orientalis* and *M. sieversii* from *M. domestica* (mean proportions of ancestry in the *M. domestica* genepool of 0.12, 0.10, 0.03 and 0.23, respectively; Table 3). Considering genotypes with membership coefficients ≥0.9 in the *M. domestica* genepool as misclassified, we found a total of *N* = 31 misclassified wild *Malus* individuals. These results suggest gene flow from the domesticated apple genepool could significantly affect the genetic integrity of wild apple relatives, their future evolution and, possibly, their use as resources for crop improvement.

### Inference of demographic history

Model-based Bayesian clustering algorithms, such as that implemented in STRUCTURE, have a high level of power only for the detection of recent introgression events [55], [61], [62]. We therefore investigated the contributions of *M. sylvestris* and *M. orientalis* to the *M. domestica* genepool using approximate Bayesian computation (ABC) methods that offer a more historical perspective on gene flow [63]. We used a demographic model implementing admixture events [64].

**Figure 4:**
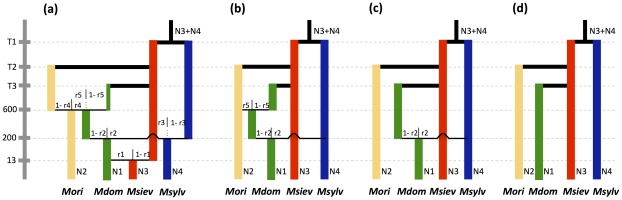
Admixture models compared in approximate Bayesian computations. Model *a* assumes that *M. domestica* is derived from *M. sieversii* and that the ancestral *M. domestica* population was involved in reciprocal introgression events with *M. orientalis* and *M. sylvestris*, and subsequently introgressed back into *M. sieversii*. Model *b* assumes no introgression from *M. domestica* into wild species, model *c* assumes the only admixture event is from *M. sylvestris* into *M. domestica*, and model *d* assumes no admixture. Admixture times between *M. domestica* and the three wild species were fixed (see text). Abbreviations: *Nk*, effective population sizes; *Tk*, divergence times; *r1*, *r3*, *r4* introgression from *M. domestica* into *M. sieversii*, *M. sylvestris*, and *M. orientalis* respectively; *r2*, *r5* introgression from *M. sylvestris* and *M. orientalis*, respectively, into *M. domestica*.

We compared several admixture models to infer what species pairs underwent introgression events and to estimate introgression rates [64]. *Malus baccata* was not included in these analyses because of its high level of divergence from *M. domestica*. We assumed, as suggested by previous studies, that *M. domestica* derived originally from *M. sieversii*. The most complex model simulated sequential admixtures between *M. domestica* and all wild species. Other models sequentially removed introgression with each wild species, the order being based on *F_ST_* values and admixture rates inferred by STRUCTURE. The compared models were the following: (i) the model *a* assumed that *M. domestica* was derived from *M. sieversii* and that the ancestral *M. domestica* population was involved in reciprocal introgression events with *M. orientalis* and *M. sylvestris*, and subsequently introgressed back into *M. sieversii* (Figure 4a), (ii) model *b* was similar to the model *a*, but without introgression events from *M. domestica* into wild species (Figure 4b), (iii) the model *c* included a single introgression event, from *M. sylvestris* into *M. domestica* (Figure 4c), and (iv) the model *d* simulated no admixture (Figure 4d). The number of parameters estimated in the model was limited by fixing the times of admixture with *M. orientalis*, *M. sylvestris* and *M. sieversii* at 600, 200 and 13 generations before the present, respectively. We used the following underlying hypotheses: (i) as the juvenile period of *Malus* lasts five to 10 years, we assumed a generation time of 7.5 years, (ii) admixture between ancestral *M. domestica* and *M. orientalis* in the Caucasus occurred approximately 4,500 years ago, shortly before the appearance of sweet apples in the Middle East (4,000 years ago), (iii) admixture between ancestral *M. domestica* and *M. sylvestris* in Europe occurred approximately 1,500 years ago, soon after the introduction of domesticated apples into Europe by the Greeks and Romans (iv) back-introgression into *M. sieversii* from *M. domestica* occurred approximately 100 years ago, when the cultivation of modern varieties reached Central Asia.

The relative posterior probabilities computed for each model provided strongest statistical support for model *c*, which assumed a single introgression event, from *M. sylvestris* into *M. domestica* (Table 4; posterior probability [*p*] = 0.67, 95% confidence interval: 0.63–0.72). Note that the model without admixture (model *d*) had the lowest relative posterior probability (Table 4). In analyses under alternative admixture models (models *a* and *b*), the posterior distributions were flat for introgression between *M. domestica* and *M. orientalis* and highly skewed towards low values for introgression into *M. sylvestris* and *M. sieversii* (not shown), which is consistent with statistical support being highest for model *c*.

**Table 4:** Relative posterior probabilities (*p*) for the four historical models compared using approximate Bayesian computations.

Model	*p*	CI2.5	CI97.5
*a*	0.0349	0.0253	0.0445
*b*	0.2819	0.2509	0.3130
*c*	0.6832	0.6504	0.7159
*d*	0.0000	0.0000	0.0000

Models are described in Figure 4. CI2.5 and CI97.5 are boundaries of the 95% confidence intervals.

Given that the model *c* was clearly favoured, parameter estimates are shown below only for this model (Table 5; prior distributions in Table S4). The contribution of *M. sylvestris* to the *M. domestica* genepool was estimated at about 61% (95% credibility interval [95% CI]: 50–68%). We obtained estimates of effective population sizes of 3,520 (95% CI: 2,090–5,680) for *M. domestica*, 13,200 (95% CI: 6,920–19,300) for *M. sieversii*, 34,600 (95% CI: 15,100–48,000) for *M. sylvestris*, and 28,300 (95% CI: 11,700–64,000) for *M. orientalis*. Using a generation time of 7.5 years, the divergence between *M. domestica* and *M. sieversii* (*T3*) was estimated to have occurred 17,700 years ago (95% CI: 6,225–25,200), which is earlier than previously thought, but we note that the credibility interval is quite large. We estimated that *M. sylvestris* and *M. sieversii* diverged about 83,250 years ago (*T1*, 95% CI: 40,575–334,500), with *M. orientalis* and *M. sieversii* diverging about 20,775 years ago (*T2*, 95% CI: 9,900–47,775).

**Table 5:** Demographic and mutation parameters estimated using approximate Bayesian computation for model *c*.

Parameter	Mode	CI2.5	CI97.5
*N1* (*M. dom*)	3,520	2,090	5,680
*N2* (*M. ori*)	28,300	11,700	64,000
*N3* (*M. siev*)	13,200	6,920	19,300
*N4* (*M. sylv*)	34,600	15,100	48,000
*T1* (*M. siev* - *M.sylv*)	11,100	5,410	44,600
*T2* (*M. siev* - *M. ori*)	2,770	1,320	6,370
*T3* (*M. siev* - *M. dom*)	2,360	830	3,360
*r2* (introgr. by *M. sylv* into *M. dom*)	0.61	0.50	0.68
*μ*	2.0.10^−4^	1.1.10^−4^	6.9.10^−4^
*p*	0.3	0.1	0.3
*μSNI*	3.0.10^−8^	5.0.10^−8^	5.9.10^−5^
*θ1* ( = 4*N1μ*)	0.7	0.5	2.5
*θ2* ( = 4*N2μ*)	6.1	3.1	23.6
*θ3* ( = 4*N3μ*)	2.8	1.9	7.4
*θ4* ( = 4*N4μ*)	6.8	4.4	18.1
*τ1* ( = *μT1*)	2.28	1.30	16.10
*τ2* ( = *μT2*)	0.54	0.31	2.38
*τ3* ( = *μT3*)	0.41	0.19	1.55

Posterior distributions are summarized as the mode and boundaries of the 95% credibility intervals (CI2.5 and CI97.5). Demographic parameters are introduced in Figure 4 (note that admixture times are fixed in these analyses). Composite parameters scaled by the mutation rate are also shown. The mutation parameters are *μ* (mean mutation rate), *p* (mean value of the geometric distribution parameter that governs the number of repeated motifs that increase or decrease the length of the locus during mutation events), *μSNI* (mean single nucleotide indel mutation rate). Species names are abbreviated.

The results above were obtained using the full dataset. We checked the validity of our inferences by conducting analyses on the dataset without admixed and misclassified individuals and using different times of admixture, by assessing the goodness-of-fit of models to data, and by checking that sufficient power was achieved to discriminate among competing models (Text S1; Tables S5, S6, S7). Overall, ABC analyses all provided clear support for a model with contribution of the European crabapple into the domesticates, although the estimated value of the actual contribution of *M. sylvestris* is probably overestimated here, and should therefore be treated with caution. Indeed, the simulation of a single introgression event hundreds of years ago most likely demanded higher rates of introgression to account for the actual genetic contribution of *M. sylvestris* into *M. domestica* than would be needed under continuous gene flow over a long period.

### Weak genetic structure within *M. domestica*: linked to cultivar use or geography?

**Figure 5:**
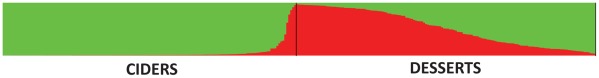
Proportions of ancestry of *M. domestica* genotypes (cider and dessert apples) in two ancestral genepools inferred with the STRUCTURE program.

As cider cultivars produce apples that are smaller, more bitter and astringent than dessert cultivars, we expected to observe genetic differentiation between these two groups of cultivars and a closer genetic proximity of cider cultivars to *M. sylvestris* [35], [65]. Neither hypothesis was supported by our data. The classification of apples into “dessert” and “cider” varieties as prior information for STRUCTURE (*Locprior* model) revealed a very weak tendency of cider and dessert cultivars to be assigned to different clusters at *K* = 2 (Figure 5), but increasing *K* did not further result in clearer differentiation between the two types of cultivars. At *K* = 2, *M. domestica* cider genotypes had a mean membership of 94.7%, and *M. domestica* dessert genotypes had a mean membership of 52.5%. However, STRUCTURE analyses without this prior information gave essentially the same clustering patterns at *K* = 2 (*G′* = 0.95 similarity to analyses using classification to assist clustering). The weak differentiation between cider and dessert cultivars (*F_ST_* = 0.02) and their high level of admixture in STRUCTURE analyses (Figure 5) indicated a shallow subdivision of the *M. domestica* genepool. Analyses on a pruned dataset from which closely related individuals had been removed (*i.e.*, pairs of genotypes with *r_xy_*≥0.5; *N* = 172) revealed the same pattern, confirming that the presence of related cultivars in the dataset did not bias clustering analyses. STRUCTURE was also run on a dataset including all *M. sylvestris* genotypes, to test the hypothesis that cider cultivars would display a higher level of introgression from the European crabapple. However, the opposite pattern was observed: the proportion of genotypes displaying introgression from *M. sylvestris* was actually significantly higher in dessert than in cider cultivars (36.4% and 15.5% respectively, *χ^2^* = 16.9, *P* = 4×10^−5^). Finally, little genetic differentiation was observed between groups of cultivars of different geographic origins (95% CI: −0.8–0.6, Table S8).

## Discussion

The apple is so deeply rooted in the culture of human populations from temperate regions that it is often not recognized as an exotic plant of unclear origin. We show here that the evolution of the domesticated apple involved more than one geographically restricted wild species. The domesticated apple did not arise from a single event over a short period of time, but from evolution extending over thousands of years. The genepool of the current domesticated apple varieties has been enriched by the contribution of at least two wild species. *Malus* species have a self-incompatibility system; apple domestication and traditional variety improvement have therefore been based mostly on the selection of the best phenotypes grown from open-pollinated seeds. This breeding strategy has probably favoured the incorporation of genetic material from multiple wild sources and the maintenance of high levels of genetic variation in domesticated apples, despite the extensive use of large-scale vegetative propagation of superior individuals by grafting. Our results are consistent with those reported for the few other woody perennials studied to date, such as grape [37], red mombin [26] and olive trees [36], and support the view that domestication in long-lived plants differs in many respects from the scenarios described for seed-propagated annuals.

### Weak differentiation from wild progenitors and the Central Asian origin of *M. domestica*

*Malus sieversii* was previously identified as the main contributor to the *M. domestica* genome on the basis of morphological and sequence data [41], [43]. The flanks of the Tian Shan mountains have been identified as a likely initial site of domestication, based on the high morphological variability of the wild apples growing in this region, and their similarity to sweet dessert apples [44], [45]. We show here, using a set of rapidly evolving genetic markers distributed throughout the genome and a large sampling, that *M. domestica* now forms a distinct, random mating group, surprisingly well separated from *M. sieversii*, with no difference in levels of genetic variation between the domesticate and its wild progenitor. This contrasts with the pattern previously reported, based on a twenty three-gene phylogenetic network [43], where domesticated varieties of apple appeared nested within *M. sieversii*. After the removal of individuals showing signs of recent admixture, *M. sieversii* and *M. domestica* nevertheless appeared to be the pair of species most closely related genetically, confirming their progenitor-descendant relationship.

### Lack of a domestication bottleneck

Apple breeding methods (grafting and “chance seedling” selection), life-history traits specific to trees and/or the genetic architecture of selected traits have likely played a role in the conservation of levels of genetic diversity in cultivated apples similar to those in wild apples. Some factors, such as “chance seedling” selection [66], may even have increased genetic diversity, by favouring outcrossing events among domesticates and introgression from wild species [39]. The low inbreeding coefficients inferred in domesticated apples and the low level of differentiation between cultivated and wild apple populations [40], [54], [67], [68] indicate a high frequency of crosses between individuals of *M. domestica*, *M. sieversii* and other wild relatives hailing from diverse geographic origins. Such a high level of gene flow has likely contributed to maintenance of a high level of genetic diversity in domesticated apples.

The grafting technique, which was probably developed around 3,000 years ago, has made it possible to propagate superior individuals clonally. The spread of grafting, together with the lengthy juvenile phase (5–10 years) and the long lifespan of apples, may have imposed strong limits on the intensity of the domestication bottleneck thereby limiting the loss of genetic diversity [27], [28], [31]. By decreasing the number of generations since domestication, these factors have probably also helped to restrict the differentiation between domesticates and wild relatives. In theory, grafting may have limited the size of the apple germplasm dispersed early on to a few very popular genotypes, thereby provoking a sudden shrink in effective population size and a loss of diversity. However, we found no evidence that the clonal propagation of apples resulted in a long-lasting decrease in population size or clonal population structure. We can speculate that this may be due to a combination of various factors such as: gene flow with wild species, small-scale propagation (many farmers producing a few grafts each), a large variation in preferences for taste and other quality characteristics between farmers and cultures, large differences in growth conditions leading to the adoption of different sets of genotypes in different regions or the typical behaviour of hobby breeders, who tend to spot particular differences and multiply them. Similarly, for grape, there are huge numbers of old varieties and as much genetic variation in cultivated varieties as in wild-relative progenitors [37].

### A major secondary contribution from the European crabapple

There has been a long-running debate concerning the possible contribution of other wild species present along the Silk Route to the genetic makeup of *M. domestica* [40], [46], [47], [65], [69]. Our results clearly show that interspecific hybridization has been a potent force in the evolution of domesticated apple varieties. Apple thus provides a rare example of the evolution of a domesticated crop over a long period of time and involving at least two wild species (see also the cases of olive tree and avocado [24], [26], [37], [70]). A recent study argued that introgression from *M. sylvestris* into the *M. domestica* genepool was the most parsimonious explanations for shared gene sequence polymorphisms between the two species [50]. Using an unprecedentedly large dataset, more numerous and more rapidly evolving markers and a combination of inferential methods, we provide a comprehensive view of the history of domestication in apple. We confirm that *M. sieversii* was the initial progenitor and show that the wild European crabapple *M. sylvestris* has been a major secondary contributor to the diversity of apples, resulting in current varieties of *M. domestica* being more closely related to *M. sylvestris* than to their central Asian progenitor. This situation is reminiscent of that for maize, in which the cultivated crop *Zea mays* is genetically more closely related to current-day highland landraces than to lowland *Z. mays* ssp. *parviglumis* from which the crop was domesticated [71]. This pattern has been attributed to large-scale gene flow from a secondary source, a second subspecies of teosinte, *Z. mays* ssp. *mexicana*, into highland maize populations [71].

The usefulness of wild relatives for improving elite cultivated crop genepools has long been recognised and the exploitation of wild resources is now considered a strategic priority in breeding and conservation programs for most crops [11], [12], [44]. Domesticated apples are unusual in that the contribution of wild relatives probably occurred early and unintentionally in the domestication process, preceding even the use of controlled crosses. The use of genetic markers with lower mutation rates than our set of microsatellites might also make it possible to investigate the contribution of more phylogenetically distant apple species growing in areas away from the Silk Route to the diversification of modern apple cultivars.

The Romans introduced sweet apples into Europe at a time at which the Europeans were undoubtedly already making cider from the tannin-rich fruits of the native *M. sylvestris* [35], [72]. Cider is not typical of Asia [35], but it was widespread in Europe by the time of Charlemagne (9^th^ century, [73]). Large numbers of apple trees were planted for cider production in France and Spain from the 10^th^ century onwards [48], [52]. The very high degree of stringency of cider apples (often to the extent that they are inedible) led to the suggestion that cider cultivars arose from hybridization between *M. sylvestris* and sweet apples [35], [46], [65]. We show here that the genetic structure within the cultivated apple genepool is very weak, with poor differentiation between cider and dessert apples. Cider cultivars thus appear to be no more closely genetically related to *M. sylvestris* than dessert cultivars. As wild Asian apples are known to cover the full range of tastes [44], [46], it is possible that fruits with the specific characteristics required for cider production were in fact initially selected in Central Asia and subsequently brought into Europe. There is a long-standing tradition of cider production in some parts of Turkey [35], for instance, which is potentially consistent with an Eastern origin of cider cultivars. However, the low level of genetic differentiation between dessert and cider apples indicates that, even if different types of apples were domesticated in Asia and brought to Europe, they have not diverged into independent genepools.

### Concluding remarks

This study settles a long-running debate by confirming that 1) *M. domestica* was initially domesticated from *M. sieversii*, and 2) *M. domestica* subsequently received a significant genetic contribution from *M. sylvestris*, much larger than previously suspected [35], at least in Western Europe, where originated most of our samples and most cultivar diversity. The higher level of introgression of the European crabapple into the domesticated apple in this study than in previous studies [43], [50], [51] may be attributed to the use of a larger and more representative set of *M. domestica* genotypes coupled with the genotyping of numerous and rapidly evolving markers known to trace back more recent events.

Our inferences also have important implications for breeding programs and for the conservation of wild species of apple. The major contribution of the various wild species to the *M. domestica* genepool highlights the need to invest efforts into the conservation of these species, which may contain unused genetic resources that could further improve the domesticated apple germplasm [74], such as disease resistance genes or genes encoding specific organoleptic features.

## Materials and Methods

### Sample collection and DNA extraction

Leaf material was retrieved from the collections of various institutes (INRA Angers, France; USDA - ARS, Plant Genetic Resources Unit, Geneva, NY; ILVO Melle, Belgium) and from a private apple germplasm repository in Brittany for *M. domestica* (*N* = 368, Figure S1 including only diploid cultivars *N* = 299) and from forests for the four wild species (Figure 1; Table S1). *Malus sieversii* (*N* = 168) material was collected from 2007 to 2010 in the Chinese Xinjiang province (*N* = 26), Kyrgyzstan (*N* = 5), Uzbekistan (*N* = 1), Tajikistan (*N* = 1) and Kazakhstan (*N* = 114). *Malus orientalis* (*N* = 215) was sampled in 2009 in Armenia (*N* = 203), Turkey (*N* = 5) and Russia (*N* = 5). *Malus sylvestris* (*N* = 40) samples were obtained from 15 European countries. *Malus baccata* (*N* = 48) was sampled in 2010 in Russia. The origins of *M. domestica* cultivars were: France (*N* = 266), Great Britain (*N* = 12), USA (*N* = 12), Russia (*N* = 7), the Netherlands (*N* = 6), Australia (*N* = 4), Belgium (*N* = 4), Germany (*N* = 4), Japan (*N* = 3), Ukraine (*N* = 3), Tunisia (*N* = 2), Switzerland (*N* = 2), Spain (*N* = 2), New Zealand (*N* = 2), Israel (*N* = 1), Ireland (*N* = 1), Canada (*N* = 1), Armenia (*N* = 2) and unknown/debated (*N* = 34). Genomic DNA was extracted with the Nucleo Spin plant DNA extraction kit II (Macherey & Nagel, Düren, Germany) according to the manufacturer's instructions.

### Microsatellite markers and polymerase chain reaction (PCR) amplification

Microsatellites were amplified by multiplex PCR, with the Multiplex PCR Kit (QIAGEN, Inc.). We used 26 microsatellites spread across the 17 chromosomes (one to three microsatellites per chromosome), in 10 different multiplexes previously optimised on a large set of genetically related progenies of *M. domestica* [75]. The four multiplexes (MP01, MP02, MP03, MP04; Table S9; Lasserre P. unpublished data) were performed in a final reaction volume of 15 µl (7.5 µl of QIAGEN Multiplex Master Mix, 10–20 µM of each primer, with the forward primer labelled with a fluorescent dye and 10 ng of template DNA). We used a touch-down PCR program (initial annealing temperature of 60°C, decreasing by 1°C per cycle down to 55°C). Six other multiplex reactions (Hi6, Hi4a^b^, Hi5-10, Hi13a, Hi13b, Hi4b) were performed using previously described protocols [75]. Genotyping was performed on an ABI PRISM X3730XL, with 2 µl of GS500LIZ size standard (Applied Biosystems). Alleles were scored with GENEMAPPER 4.0 software (Applied Biosystems). We retained only multilocus genotypes presenting less than 30% missing data.

### Suitability of microsatellites for population genetic analyses

We checked the suitability of the markers for population genetic analyses. None of the 26 microsatellite markers deviated significantly from a neutral equilibrium model, as shown by the non significant *P*-values obtained in Ewen-Watterson tests [76], and no pair of markers was found to be in significant linkage disequilibrium in any of the species [77], [78]. The markers could therefore be considered unlinked and neutral.

### Analyses of genetic variation and differentiation between the five species

Apple cultivars may be polyploid [79]. We therefore first checked for the presence of polyploidy individuals of *M. domestica* within our dataset. Individuals presenting multiple peaks on electrophoregrams were first re-extracted to eliminate contamination as a possible source of apparent polyploidy. We then checked whether they had been reported to be polyploidy in previous studies [79]. After completion of this checking procedure, we removed 69 polyploids (of the 368 samples) from subsequent analyses. We tested for the occurrence of null alleles at each locus with MICROCHECKER 2.2.3 software [80]. Allelic richness and private allele frequencies were calculated with ADZE software [81], for a sample size of 22. Heterozygosity (expected (*H_E_*) and observed (*H_O_*)), Weir & Cockerham *F*-statistics, deviation from Hardy-Weinberg equilibrium and genotypic linkage disequilibrium were estimated with GENEPOP 4.0 [77], [78]. The significance of differences between *F_ST_* values was assessed in exact tests carried out with GENEPOP 4.0 [77], [78]. Individuals were assigned to clonal lineages with GENODIVE [53]. We estimated relatedness between pairs of cultivars and between pairs of individuals within each species, by calculating the *r_xy_* of Ritland and Lynch [82] with RE-RAT online software [83]. We tested whether the distributions of *r_xy_* deviated significantly from a Gaussian distribution with a mean of zero and a standard deviation equal to the observed standard deviation, by comparing observed and simulated distributions in Fisher's exact test (R Development Core Team, URL http://www.R-project.org).

### Assessing bottlenecks during apple domestication and diversification

We tested for the occurrence of a bottleneck during apple domestication with the method implemented in BOTTLENECK [58], [84]. The tests were performed under the stepwise-mutation model (SMM) and under a two-phase model (TPM) allowing for 30% multistep changes. We used Wilcoxon signed rank tests to determine whether a population had a significant number of loci with excess genetic diversity.

### Analyses of population subdivision

We used the individual-based Bayesian clustering method implemented in STRUCTURE 2.3.3 [55], [85], [86] to investigate species delimitation, intraspecific population structure and admixture. This method is based on Markov Chain Monte Carlo (MCMC) simulations and is used to infer the proportion of ancestry of genotypes in *K* distinct predefined clusters. The algorithm attempts to minimize deviations from Hardy–Weinberg and linkage equilibrium within clusters. Analyses were carried out without the use of prior information, except for analyses of population subdivision within the *M. domestica* genepool for which the “cider”/“dessert” classification of cultivars was used as prior information to assist clustering. *K* ranged from 1 to 8 for analyses of the five-species dataset and the *M. domestica* dataset, and was fixed at *K* = 2 for analyses of pairs of species including *M. domestica* and each of the wild species. Ten independent runs were carried out for each *K* and we used 500,000 MCMC iterations after a burn-in of 50,000 steps. We used CLUMPP v1.1.2 (Greedy algorithm) [87] to look for distinct modes among the 10 replicated runs of each *K*.

STRUCTURE analyses were run for the full dataset (*N* = 839) and for two pruned datasets excluding non-pure individuals (*i.e.*, genotypes with <0.9 membership of their species' genepool) and related individuals (*r_xy_*≥0.5).

### Inference of demographic history

We used the DIYABC program [88] to compare different admixture models and infer historical parameters. We simulated microsatellite datasets for 14 loci (Ch01h01, Ch01h10, Ch02c06, Ch02d08, Ch05f06, Ch01f02, Hi02c07, Ch02c09, Ch03d07, Ch04c07, Ch02b03b, MS06g03, Ch04e03, Ch02g01) previously reported to be of the perfect repeat type [89], [90], [91]. In total, we generated 5×10^5^ simulated datasets for each model.

A generalized stepwise model (GSM) was used as the mutational model. The model had two parameters: the mean mutation rate (*μ*) and the mean parameter (*P*) of the geometric distribution used to model the length of mutation events (in numbers of repeats). As no experimental estimate of microsatellite mutation rate is available for *Malus*, the mean mutation rate was drawn from a uniform distribution by extreme values of 10^−4^ and 10^−3^, and the mutation rate of each locus was drawn independently from a Gamma distribution (mean = *μ*; shape = 2). The parameter *P* ranged from 0.1 to 0.3. Each locus L had a possible range of 40 contiguous allelic states (44 for *CH02C06*, 42 for *CH04E03*) and was characterized by individual values for mutation rate (*μ_L_*) and the parameter of the geometric distribution (*P_L_*); *μ_L_* and *P_L_* were drawn from Gamma distributions with the following parameter sets: mean = *μ*, shape = 2, range = 5×10^−5^–5×10^−2^ for *μ_L_*, and mean = *P*, shape = 2, range = 0.01–0.9 for *P_L_*. As not all allele lengths were multiples of motif length, we also included single-nucleotide insertion-deletion mutations in the model, with a mean mutation rate (*μ_SNI_*) and locus-specific rates drawn from a Gamma distribution (mean = *μ_SNI_*; shape = 2). The summary statistics used were: mean number of alleles per locus, mean genetic diversity [92], genetic differentiation between pairwise groups (*F_ST_*; [93]), genetic distances (*δμ*)^2^ [94].

We used a polychotomous logistic regression procedure [95] to estimate the relative posterior probability of each model, based on the 1% of simulated data sets closest to the observed data. Confidence intervals for the posterior probabilities were computed using the limiting distribution of the maximum likelihood estimators [64]. Once the most likely model was identified, we used a local linear regression to estimate the posterior distributions of parameters under this model [96]. The 1% simulated datasets most closely resembling the observed data were used for the regression, after the application of a *logit* transformation to parameter values.

## Supporting Information

Figure S1 Geographic origins of diploid *M. domestica* cultivars (*N* = 299). See details in Table S1.
(TIF)

Figure S2 Distribution of pairwise relatedness coefficients [82] among the *M. domestica* cultivars. *r_xy_* values among cultivars are normally distributed around a mean of zero, with a low variance between pairs of cultivars (Fisher's exact test, *P*≈1).
(TIF)

Figure S3 Proportions of ancestry in two ancestral genepools inferred with the STRUCTURE program from datasets including *M. domestica* (green, *N* = 89) and each of the wild *Malus* species (red) except *M. baccata*. The x-axis is not at scale.
(TIF)

Table S1 Description of the *Malus* species accessions analysed, with their geographic origin and providers.
(DOC)

Table S2 *Malus domestica* cultivars used in the study, with their use, provider, geographic putative origin. Details of the STRUCTURE analysis summarized in Table 3 are also provided.
(DOC)

Table S3 Membership coefficients inferred from the STRUCTURE analysis for *M. baccata* individuals.
(DOC)

Table S4 Prior distributions used in approximate Bayesian computations. Prior distributions are uniform between lower and upper bound. Parameters are introduced in Figure 4 and Table 5. Species names are abbreviated.
(DOC)

Table S5 Relative posterior probabilities (*p*) for the four historical models compared using approximate Bayesian computations. Models are described in Figure 4. CI2.5 and CI97.5 are boundaries of the 95% confidence intervals. (A) Analyses on a pruned dataset with misclassified wild individuals and individuals with a recent admixed ancestry removed. (B) Analyses on the full dataset, assuming that admixture between ancestral *M. domestica* and *M. sylvestris* was more recent (67 generations–500 ybp) than in original analyses (200 generations–1,500 ybp).
(DOC)

Table S6 Demographic and mutation parameters estimated using approximate Bayesian computation for model *c*. Posterior distributions are summarized as the mode and boundaries of the 95% credibility intervals (CI2.5 and CI97.5). Demographic parameters are introduced in Figure 4 (note that admixture times are fixed in these analyses). Composite parameters scaled by the mutation rate are also shown. The mutation parameters are *μ* (mean mutation rate), *p* (mean value of the geometric distribution parameter that governs the number of repeated motifs that increase or decrease the length of the locus during mutation events), *μSNI* (mean single nucleotide indel mutation rate). Species names are abbreviated. (A) Analyses on a pruned dataset with misclassified wild individuals and individuals with a recent admixed ancestry removed. (B) Analyses on the full dataset, assuming that admixture between ancestral *M. domestica* and *M. sylvestris* was more recent (67 generations–500 ybp) than in original analyses (200 generations–1,500 ybp).
(DOC)

Table S7 Model checking based on comparisons of test quantities between observed data and 100 pseudo-observed datasets generated using parameter values drawn from posterior distributions. (A) Analyses on the full dataset, (B) Analyses on a pruned dataset with misclassified wild individuals and individuals with a recent admixed ancestry removed. (C) Analyses on the full dataset, assuming that admixture between ancestral *Malus domestica* and *M. sylvestris* was more recent (67 generations–500 ybp) than in (A).
(DOC)

Table S8 Genetic differentiation (*F_ST_*) between cultivars of different geographic origins (*N* = 266). Cultivars of unknown origin have been removed.
(DOC)

Table S9 Description of the Multiplex PCRs (MP01, MP02, MP03, MP04) used for microsatellite amplification.
(DOC)

Text S1 Method used for approximate Bayesian computations on alternative datasets/admixture times.
(DOC)
