# Supplementary figures and images for: New Insight into the History of Domesticated Apple: Secondary Contribution of the European Wild Apple to the Genome of Cultivated Varieties
Source: PLoS Genet. 2012 May 10;8(5):e1002703. doi: 10.1371/journal.pgen.1002703 (PMC3349737; doi:10.1371/journal.pgen.1002703)

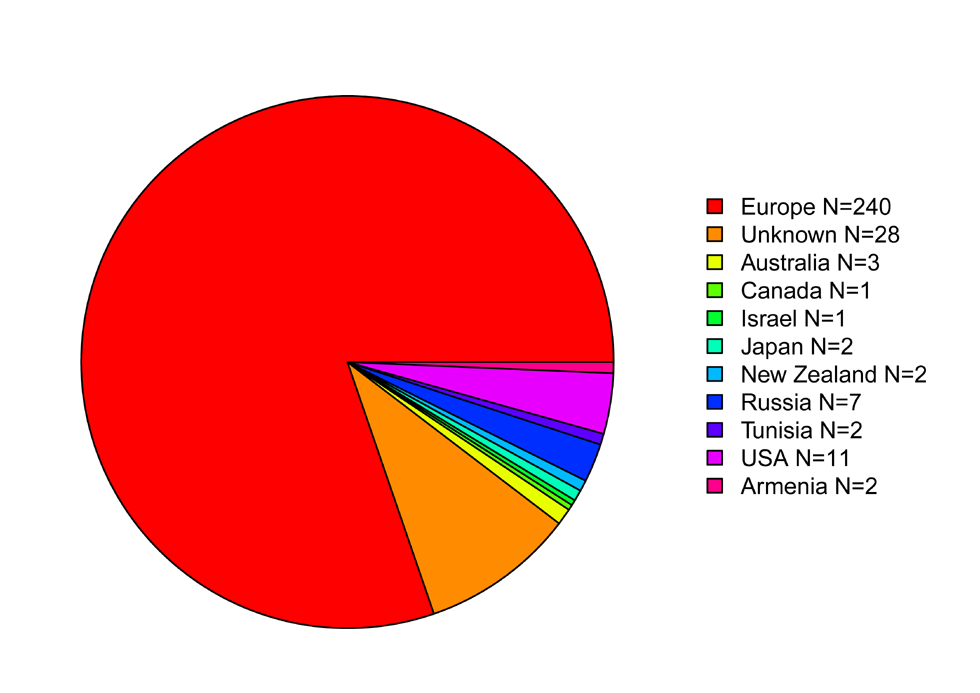

Supplement: Figure S1 — Geographic origins of diploid M. domestica cultivars (N = 299). See details in Table S1. (TIF) [file pgen.1002703.s001.tif]

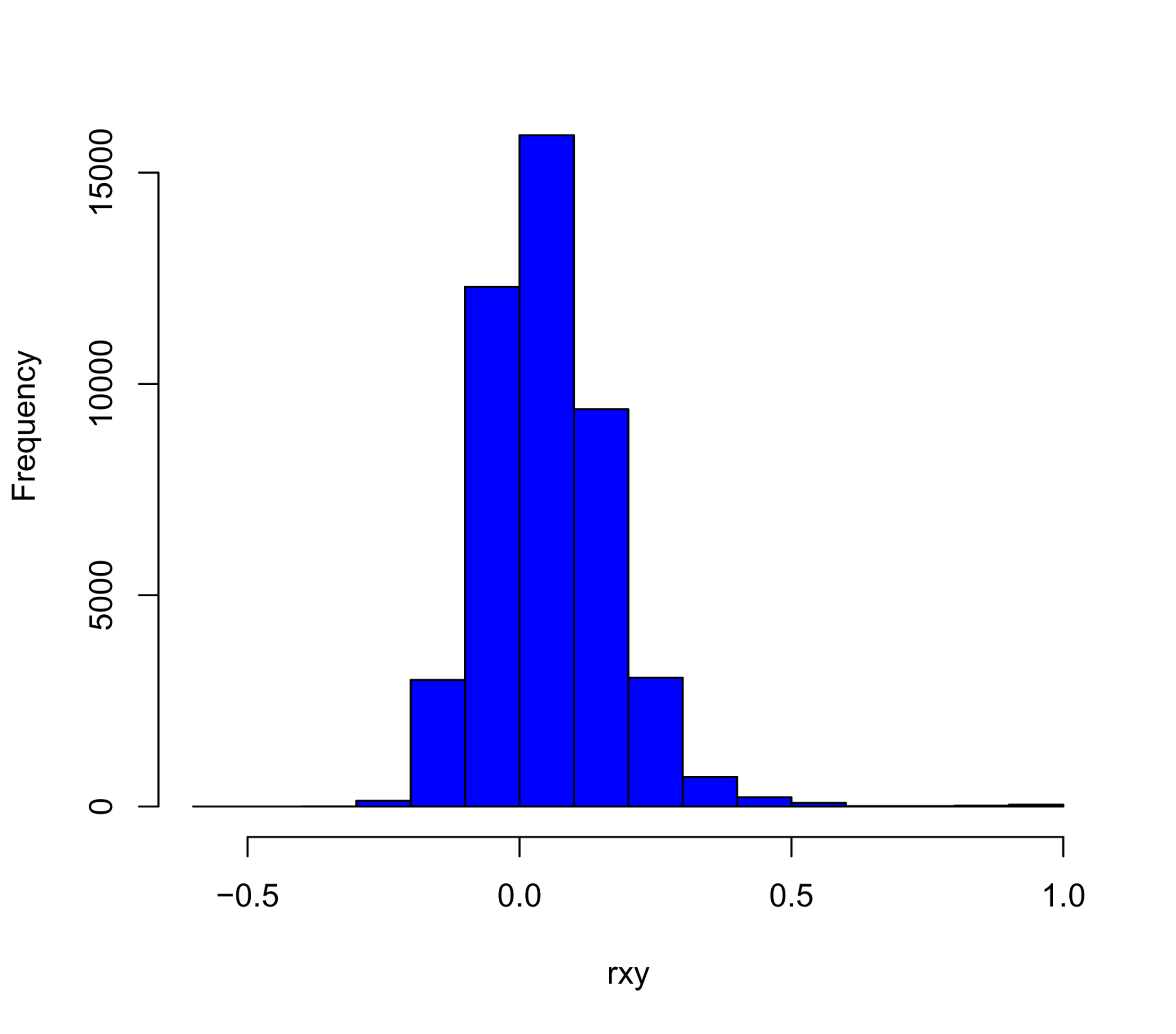

Supplement: Figure S2 — Distribution of pairwise relatedness coefficients [82] among the M. domestica cultivars. rxy values among cultivars are normally distributed around a mean of zero, with a low variance between pairs of cultivars (Fisher's exact test, P≈1). (TIF) [file pgen.1002703.s002.tif]

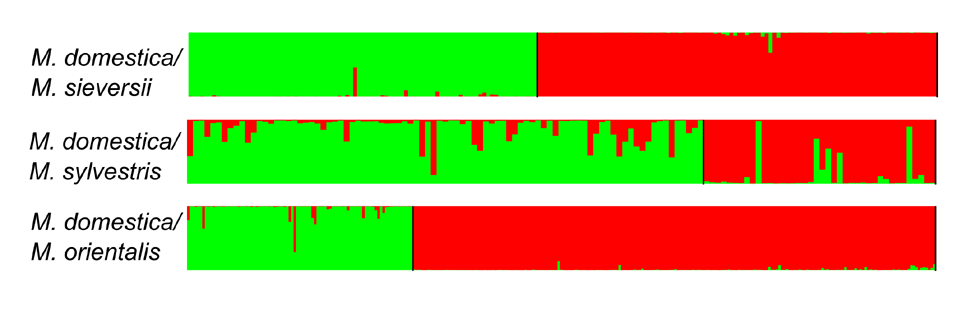

Supplement: Figure S3 — Proportions of ancestry in two ancestral genepools inferred with the STRUCTURE program from datasets including M. domestica (green, N = 89) and each of the wild Malus species (red) except M. baccata. The x-axis is not at scale. (TIF) [file pgen.1002703.s003.tif]
