# Supplementary material for: New Insight into the History of Domesticated Apple: Secondary Contribution of the European Wild Apple to the Genome of Cultivated Varieties
Source: PLoS Genet. 2012 May 10;8(5):e1002703. doi: 10.1371/journal.pgen.1002703 (PMC3349737; doi:10.1371/journal.pgen.1002703)
Supplement: Table S1 — Description of the Malus species accessions analysed, with their geographic origin and providers. (DOC) [file pgen.1002703.s004.doc]

| Table S1. Description of the *Malus* species accessions analysed, with their geographic origin and providers. | | |
| --- | --- | --- |
|  |  |  |
| Species | Nb* | Provider |
| *Malus sylvestris* | 40 |  |
| Austria | 2 | Thomas and Bernhard Kirisits |
| Belgium | 4 | CRA-W^1^, IVLO^2^ |
| Bosnia-Herzegovina | 3 | Dalibor Ballian |
| Bulgaria | 1 | Petya Gercheva, Argir Zhivondov,Valentina Bojkova, Anna Matova |
| Danemark | 6 | Anders Larsen |
| France | 5 | INRA^3^, USDA-ARS^4^ |
| Germany | 5 | Jorg Kleinschmit and Wilfried Steiner |
| UK, Scotland | 2 | Carvers Stephens |
| Hungary | 2 | Lazlo Nyari and Heino Konrad |
| Italy | 2 | Alberto Dominicci |
| Norway | 2 | Per Avid |
| Poland | 2 | Jan Kowalsky et Dzmitry Kahan |
| Romania | 1 | Lucian Curtus |
| Spain | 2 | Carlos Ferrera et Francisco Donaire |
| Ukraine | 1 | Roman Volansyanchuk |
| *Malus sieversii* | 168 |  |
| Kazakhstan | 114 | Field sampling |
|  | 28 | USDA-ARS^4^ |
| China | 26 | Xinjiang |
| Kirghizistan | 5 | Field sampling |
| Tadjikistan | 1 | USDA-ARS^4^ |
| Uzbekistan | 1 | USDA-ARS^4^ |
| *Malus orientalis* | 215 |  |
| Armenia | 203 | field sampling |
| Russia | 5 | USDA-ARS^4^ |
| Turkey | 5 | USDA-ARS^4^ |
| Unknown | 2 | USDA-ARS^4^ |
| *M. baccata* | 48 |  |
| *Unknown* | 10 | USDA-ARS^4^, EMR^6^ , IVLO^2^ |
| Russia (Transbaikal Region) | 36 | Marina Olonova, Ilya Zakarov, Natalia Badmayeva, Irina Kreshchenok |
| Romania | 1 | IVLO^2^ |
| Hungary | 1 | IVLO^2^ |
| *Malus domestica* | 368 | INRA^3^, CRA-W^1^, USDA-ARS^4^, Abbaye de Beauport^5^, Verger Conservatoire d’Arzano^7^ |
| **Diploid** | 299 |  |
| Unknown | 28 | INRA^3^, CRA-W^1^, USDA-ARS^4^ |
| Australia | 3 | INRA^3^ |
| Belgium | 4 | INRA^3^, CRA - W^1^ |
| Canada | 1 | INRA^3^ |
| France | 209 | INRA^3^, CRA-W^1^, Abbaye de Beauport^5^, Verger Conservatoire d’Arzano^7^ |
| Germany | 4 | INRA^3^, CRA - W^1^ |
| Great Britain | 9 | INRA^3^, CRA - W^1^ |
| Ireland | 1 | USDA-ARS^4^ |
| Israel | 1 | INRA^3^ |
| Japan | 2 | INRA^3^ |
| Netherland | 6 | INRA^3^, USDA-ARS^4^ |
| New Zeland | 2 | INRA^3^ |
| Russia | 7 | INRA^3^, USDA-ARS^4^ |
| Spain | 2 | INRA^3^ |
| Switzerland | 2 | INRA^3^ |
| Tunisia | 2 | INRA^3^ |
| Ukraine | 3 | INRA^3^, CRA-W^1^ |
| USA | 11 | INRA^3^ |
| Armenia | 2 | Field sampling |
| **Triploid** | **69** |  |
| Unknown | 6 | INRA^3^ |
| Australia | 1 | INRA^3^ |
| France | 58 | INRA^3^, Abbaye de Beauport^5^, Verger Conservatoire d’Arzano^7^ |
| Great Britain | 2 | INRA^3^ |
| Japan | 1 | INRA^3^ |
| USA | 1 | INRA^3^ |
| *Malus domestica* |  |  |
| Cider | 119 |  |
| Desserts | 180 |  |
|  |  |  |
|  |  |  |
| *Number of trees sampled |  |  |
| ^1^ CRA - W | Centre Wallons de Recherches Agronomiques, Belgium | |
| ^2^ IVLO - PLANT | ILVO, Plant – Growth and Development, Caritasstraat 21, 9090 Melle, Belgium | |
| ^3^ INRA | Institut National de la Recherche Agronomique, France | |
| ^4^ USDA - ARS | United States Departement of Agriculture - Agricultural Research Service, Cornell University, USA | |
| ^5^ Abbaye de Beauport | Conservatory Orchards of ancient apple varieties, Paimpol, France. | |
| ^6^ EMR | East Malling Researh, Kent, UK | |
| ^7^Verger Conservatoire d’Arzano | Conservatory Orchards of ancient apple varieties, Brittany, France. | |
