# Supplementary material for: New Insight into the History of Domesticated Apple: Secondary Contribution of the European Wild Apple to the Genome of Cultivated Varieties
Source: PLoS Genet. 2012 May 10;8(5):e1002703. doi: 10.1371/journal.pgen.1002703 (PMC3349737; doi:10.1371/journal.pgen.1002703)
Supplement: Table S2 — Malus domestica cultivars used in the study, with their use, provider, geographic putative origin. Details of the STRUCTURE analysis summarized in Table 3 are also provided. (DOC) [file pgen.1002703.s005.doc]

Table S2. *Malus domestica* cultivars used in the study, with their use, provider, geographic putative origin. Details of the STRUCTURE analysis summarized in Table 3 are also provided.

| Sample | Use | Geographical Origin | Provider | *M. domestica* | *M. sylvestris* | *M. domestica* | *M. baccata* | *M. domestica* | *M.sieversii* | *M. domestica* | *M. orientalis* |
| --- | --- | --- | --- | --- | --- | --- | --- | --- | --- | --- | --- |
| borovitsky | dessert | Russia | INRA | 0.067 | 0.933 | 0.001 | 0.999 | 0.94 | 0.06 | 0.669 | 0.331 |
| reinette ananas | dessert | ? | INRA | 0.98 | 0.02 | 0.004 | 0.996 | 0.997 | 0.003 | 0.998 | 0.002 |
| udarre zagarra | dessert | France | INRA | 0.887 | 0.113 | 0.102 | 0.898 | 0.964 | 0.036 | 0.984 | 0.016 |
| usta gorria | dessert | France | INRA | 0.628 | 0.352 | 0.001 | 0.999 | 0.956 | 0.044 | 0.912 | 0.088 |
| orleans | dessert | Netherland | INRA | 0.983 | 0.017 | 0.001 | 0.999 | 0.996 | 0.004 | 0.998 | 0.002 |
| camuesa verde | dessert | Spain | INRA | 0.585 | 0.415 | 0.049 | 0.951 | 0.942 | 0.058 | 0.993 | 0.007 |
| saint-bernard d29 | dessert | France | CRA - W | 0.934 | 0.066 | 0.001 | 0.999 | 0.997 | 0.003 | 0.996 | 0.004 |
| purpurroter cousinot c21 | dessert | Germany | CRA - W | 0.966 | 0.034 | 0.003 | 0.997 | 0.997 | 0.003 | 0.997 | 0.003 |
| pigeonnet a12 | dessert | France | CRA - W | 0.964 | 0.036 | 0.001 | 0.999 | 0.997 | 0.003 | 0.998 | 0.002 |
| mc intosh | dessert | Canada | INRA | 0.955 | 0.045 | 0.001 | 0.999 | 0.992 | 0.008 | 0.997 | 0.003 |
| douce de sfax | dessert | ? | INRA | 0.397 | 0.603 | 0.001 | 0.999 | 0.804 | 0.196 | 0.523 | 0.477 |
| calville duquesne b14 | dessert | Belgium | CRA - W | 0.504 | 0.496 | 0.001 | 0.999 | 0.958 | 0.042 | 0.984 | 0.016 |
| pun d'boche b56 | dessert | ? | CRA - W | 0.926 | 0.074 | 0.001 | 0.999 | 0.995 | 0.005 | 0.996 | 0.004 |
| keuleman jaune d15 | dessert | ? | CRA - W | 0.978 | 0.022 | 0.114 | 0.886 | 0.997 | 0.003 | 0.999 | 0.001 |
| reinette rouge etoilée b11 | dessert | Belgium | CRA - W | 0.669 | 0.331 | 0.002 | 0.998 | 0.994 | 0.006 | 0.993 | 0.007 |
| drap d'or a40 | dessert | France | CRA - W | 0.917 | 0.083 | 0.002 | 0.998 | 0.996 | 0.004 | 0.998 | 0.002 |
| grand alexandre a34 | dessert | Ukraine | CRA - W | 0.147 | 0.853 | 0.001 | 0.999 | 0.992 | 0.008 | 0.99 | 0.01 |
| président damseaux b17 | dessert | ? | CRA - W | 0.702 | 0.298 | 0.001 | 0.999 | 0.993 | 0.007 | 0.99 | 0.01 |
| keiing.rode d13 | dessert | ? | CRA - W | 0.984 | 0.016 | 0.012 | 0.988 | 0.997 | 0.003 | 0.998 | 0.002 |
| pladei d40 | dessert | ? | CRA - W | 0.972 | 0.028 | 0.003 | 0.997 | 0.997 | 0.003 | 0.998 | 0.002 |
| reinette de geer d22 | dessert | Belgium | CRA - W | 0.989 | 0.011 | 0.001 | 0.999 | 0.997 | 0.003 | 0.998 | 0.002 |
| maréchal d62 | dessert | ? | CRA - W | 0.981 | 0.019 | 0.352 | 0.648 | 0.997 | 0.003 | 0.998 | 0.002 |
| belle du bois c44 | dessert | ? | CRA - W | 0.987 | 0.013 | 0.001 | 0.999 | 0.997 | 0.003 | 0.999 | 0.001 |
| blanc braibant s02 | dessert | ? | CRA - W | 0.909 | 0.091 | 0.001 | 0.999 | 0.997 | 0.003 | 0.997 | 0.003 |
| **api k01** | **dessert** | **France** | **CRA - W** | **0.255** | **0.745** | **0.029** | **0.971** | **0.744** | **0.256** | **0.819** | **0.181** |
| streeping q16 | dessert | ? | CRA - W | 0.97 | 0.03 | 0.001 | 0.999 | 0.994 | 0.006 | 0.997 | 0.003 |
| belle fontaine s01 | dessert | ? | CRA - W | 0.932 | 0.068 | 0.001 | 0.999 | 0.995 | 0.005 | 0.998 | 0.002 |
| apez sagarra | dessert | France | INRA | 0.945 | 0.055 | 0.002 | 0.998 | 0.995 | 0.005 | 0.998 | 0.002 |
| rambour de himbsel k31 | dessert | ? | CRA - W | 0.68 | 0.32 | 0.001 | 0.999 | 0.991 | 0.009 | 0.997 | 0.003 |
| reinette struel k39 | dessert | ? | CRA - W | 0.857 | 0.143 | 0.003 | 0.997 | 0.997 | 0.003 | 0.998 | 0.002 |
| patte de loup | dessert | France | INRA | 0.979 | 0.021 | 0.275 | 0.725 | 0.997 | 0.003 | 0.998 | 0.002 |
| saint-omer k48 | dessert | France | CRA - W | 0.972 | 0.028 | 0.001 | 0.999 | 0.995 | 0.005 | 0.995 | 0.005 |
| type eisdener meulemans o32 | dessert | ? | CRA - W | 0.978 | 0.022 | 0.022 | 0.978 | 0.997 | 0.003 | 0.998 | 0.002 |
| franc bon pommier | dessert | France | INRA | 0.883 | 0.117 | 0.005 | 0.995 | 0.994 | 0.006 | 0.996 | 0.004 |
| idared | dessert | USA | INRA | 0.965 | 0.035 | 0.333 | 0.667 | 0.984 | 0.016 | 0.991 | 0.009 |
| golden delicious | dessert | USA | INRA | 0.976 | 0.024 | 0.027 | 0.973 | 0.997 | 0.003 | 0.998 | 0.002 |
| reinette etoilee | dessert | Netherland | INRA | 0.659 | 0.341 | 0.81 | 0.19 | 0.97 | 0.03 | 0.966 | 0.034 |
| rome beauty | dessert | USA | INRA | 0.876 | 0.124 | 0.011 | 0.989 | 0.994 | 0.006 | 0.993 | 0.007 |
| calville d'angleterre | dessert | Great Britain | INRA | 0.985 | 0.015 | 0.347 | 0.653 | 0.996 | 0.004 | 0.998 | 0.002 |
| calville blanc | dessert | Switherland/Germany | INRA | 0.946 | 0.054 | 0.845 | 0.155 | 0.998 | 0.002 | 0.998 | 0.002 |
| grand alexandre | dessert | Ukraine | INRA | 0.582 | 0.418 | 0.298 | 0.702 | 0.996 | 0.004 | 0.997 | 0.003 |
| james grieve | dessert | Scotland | INRA | 0.943 | 0.057 | 0.431 | 0.569 | 0.99 | 0.01 | 0.994 | 0.006 |
| reinette de landsberg | dessert | Germany | INRA | 0.52 | 0.48 | 0.015 | 0.985 | 0.997 | 0.003 | 0.998 | 0.002 |
| bismark | dessert | Australia | INRA | 0.44 | 0.56 | 0.326 | 0.674 | 0.992 | 0.008 | 0.975 | 0.025 |
| romarin blanc | dessert | ? | INRA | 0.793 | 0.207 | 0.002 | 0.998 | 0.996 | 0.004 | 0.99 | 0.01 |
| winesap | dessert | USA | INRA | 0.778 | 0.222 | 0.002 | 0.998 | 0.992 | 0.008 | 0.991 | 0.009 |
| cox's orange pippin | dessert | Great Britain | INRA | 0.454 | 0.546 | 0.536 | 0.464 | 0.994 | 0.006 | 0.995 | 0.005 |
| benoni | dessert | USA | INRA | 0.875 | 0.125 | 0.988 | 0.012 | 0.943 | 0.057 | 0.996 | 0.004 |
| democrat | dessert | ? | INRA | 0.628 | 0.372 | 0.999 | 0.001 | 0.993 | 0.007 | 0.994 | 0.006 |
| reinette bergamotte | dessert | Russia | INRA | 0.343 | 0.657 | 0.998 | 0.002 | 0.963 | 0.037 | 0.778 | 0.222 |
| **worcester pearmain** | **dessert** | **Great Britain** | **INRA** | **0.683** | **0.317** | **0.993** | **0.007** | **0.99** | **0.01** | **0.996** | **0.004** |
| ajmi | dessert | ? | INRA | 0.089 | 0.911 | 0.999 | 0.001 | 0.386 | 0.614 | 0.235 | 0.765 |
| newton pippin | dessert | USA | INRA | 0.985 | 0.015 | 0.997 | 0.003 | 0.996 | 0.004 | 0.998 | 0.002 |
| reine des reinettes | dessert | Germany | INRA | 0.922 | 0.078 | 0.999 | 0.001 | 0.998 | 0.002 | 0.999 | 0.001 |
| melrose | dessert | USA | INRA | 0.987 | 0.013 | 0.998 | 0.002 | 0.998 | 0.002 | 0.999 | 0.001 |
| jonathan | dessert | USA | INRA | 0.984 | 0.016 | 0.999 | 0.001 | 0.996 | 0.004 | 0.998 | 0.002 |
| millers seedling | dessert | ? | INRA | 0.965 | 0.035 | 0.999 | 0.001 | 0.997 | 0.003 | 0.998 | 0.002 |
| reinette du mans | dessert | France | INRA | 0.984 | 0.016 | 0.849 | 0.151 | 0.997 | 0.003 | 0.998 | 0.002 |
| chantecler | dessert | France | INRA | 0.967 | 0.033 | 0.991 | 0.009 | 0.986 | 0.014 | 0.997 | 0.003 |
| akane | dessert | Japan | INRA | 0.818 | 0.182 | 0.997 | 0.003 | 0.994 | 0.006 | 0.998 | 0.002 |
| chahla | dessert | Tunisia | INRA | 0.505 | 0.495 | 0.999 | 0.001 | 0.81 | 0.19 | 0.752 | 0.248 |
| aziza | dessert | Tunisia | INRA | 0.391 | 0.609 | 0.998 | 0.002 | 0.763 | 0.237 | 0.597 | 0.403 |
| fuji | dessert | Japan | INRA | 0.962 | 0.038 | 0.998 | 0.002 | 0.998 | 0.002 | 0.999 | 0.001 |
| granny smith | dessert | Australie | INRA | 0.753 | 0.247 | 0.999 | 0.001 | 0.997 | 0.003 | 0.995 | 0.005 |
| grifer | dessert | ? | INRA | 0.92 | 0.08 | 0.995 | 0.005 | 0.995 | 0.005 | 0.993 | 0.007 |
| elstar | dessert | Netherland | INRA | 0.968 | 0.032 | 0.997 | 0.003 | 0.997 | 0.003 | 0.998 | 0.002 |
| shiemer | dessert | ? | INRA | 0.643 | 0.357 | 0.999 | 0.001 | 0.869 | 0.131 | 0.946 | 0.054 |
| anna | dessert | Israel | INRA | 0.617 | 0.383 | 0.999 | 0.001 | 0.997 | 0.003 | 0.985 | 0.015 |
| reinette marbree | dessert | ? | INRA | 0.943 | 0.057 | 0.999 | 0.001 | 0.99 | 0.01 | 0.998 | 0.002 |
| gala | dessert | New-Zeland | INRA | 0.947 | 0.053 | 0.999 | 0.001 | 0.997 | 0.003 | 0.998 | 0.002 |
| falstaff | dessert | Great Britain | INRA | 0.938 | 0.062 | 0.999 | 0.001 | 0.996 | 0.004 | 0.995 | 0.005 |
| braeburn | dessert | New-Zeland | INRA | 0.99 | 0.01 | 0.998 | 0.002 | 0.997 | 0.003 | 0.998 | 0.002 |
| grenadier | dessert | Great Britain | INRA | 0.934 | 0.066 | 0.997 | 0.003 | 0.996 | 0.004 | 0.997 | 0.003 |
| pink lady | dessert | Australia | INRA | 0.979 | 0.021 | 0.999 | 0.001 | 0.996 | 0.004 | 0.998 | 0.002 |
| ariane | dessert | France | INRA | 0.93 | 0.07 | 0.994 | 0.006 | 0.983 | 0.017 | 0.991 | 0.009 |
| beauty of bath | dessert | Great Britain | INRA | 0.746 | 0.254 | 0.999 | 0.001 | 0.872 | 0.128 | 0.936 | 0.064 |
| pinova | dessert | Germany | INRA | 0.783 | 0.217 | 0.997 | 0.003 | 0.996 | 0.004 | 0.996 | 0.004 |
| belle de boskoop | dessert | Netherland | INRA | 0.982 | 0.018 | 0.986 | 0.014 | 0.997 | 0.003 | 0.998 | 0.002 |
| reinette baumann | dessert | Belgium | INRA | 0.985 | 0.015 | 0.999 | 0.001 | 0.996 | 0.004 | 0.998 | 0.002 |
| winter banana | dessert | USA | INRA | 0.986 | 0.014 | 0.999 | 0.001 | 0.998 | 0.002 | 0.999 | 0.001 |
| anisha hosta | dessert | France | INRA | 0.937 | 0.063 | 0.999 | 0.001 | 0.973 | 0.027 | 0.991 | 0.009 |
| cachao sagarra | dessert | France | INRA | 0.78 | 0.22 | 0.998 | 0.002 | 0.986 | 0.014 | 0.89 | 0.11 |
| margil | dessert | ? | INRA | 0.981 | 0.019 | 0.999 | 0.001 | 0.995 | 0.005 | 0.999 | 0.001 |
| **michelin** | **dessert** | **France** | **INRA** | **0.319** | **0.681** | **0.978** | **0.022** | **0.93** | **0.07** | **0.879** | **0.121** |
| worcester | dessert | Great Britain | INRA | 0.912 | 0.088 | 0.998 | 0.002 | 0.997 | 0.003 | 0.999 | 0.001 |
| **fiesta** | **dessert** | **Great Britain** | **INRA** | **0.701** | **0.299** | **0.999** | **0.001** | **0.993** | **0.007** | **0.997** | **0.003** |
| prima | dessert | USA | INRA | 0.963 | 0.037 | 0.999 | 0.001 | 0.992 | 0.008 | 0.998 | 0.002 |
| **M9** | **dessert** | **France** | **INRA** | **0.717** | **0.283** | **0.998** | **0.002** | **0.996** | **0.004** | **0.998** | **0.002** |
| delicious | dessert | ? | INRA | 0.983 | 0.017 | 0.998 | 0.002 | 0.998 | 0.002 | 0.999 | 0.001 |
| unamed | dessert | France | Verger Conservatoire d’Arzano, Brittany | 0.685 | 0.315 | 0.001 | 0.999 | 0.995 | 0.005 | 0.993 | 0.007 |
| trojen hir | cider | France | Verger Conservatoire d’Arzano, Brittany | 0.822 | 0.178 | 0.999 | 0.001 | 0.996 | 0.004 | 0.991 | 0.009 |
| unamed | dessert | France | Verger Conservatoire d’Arzano, Brittany | 0.559 | 0.441 | 0.996 | 0.004 | 0.994 | 0.006 | 0.994 | 0.006 |
| drap d’or à tort | dessert | France | Verger Conservatoire d’Arzano, Brittany | 0.813 | 0.187 | 0.999 | 0.001 | 0.995 | 0.005 | 0.992 | 0.008 |
| guillevic | cider | France | Verger Conservatoire d’Arzano, Brittany | 0.982 | 0.018 | 0.985 | 0.015 | 0.992 | 0.008 | 0.998 | 0.002 |
| mickellic | cider | France | Verger Conservatoire d’Arzano, Brittany | 0.715 | 0.285 | 0.998 | 0.002 | 0.976 | 0.024 | 0.993 | 0.007 |
| bacon melen cotro melen | cider | France | Verger Conservatoire d’Arzano, Brittany | 0.864 | 0.136 | 0.998 | 0.002 | 0.997 | 0.003 | 0.998 | 0.002 |
| judin | dessert | France | Verger Conservatoire d’Arzano, Brittany | 0.943 | 0.057 | 0.997 | 0.003 | 0.997 | 0.003 | 0.998 | 0.002 |
| tockec | dessert | France | Verger Conservatoire d’Arzano, Brittany | 0.681 | 0.319 | 0.998 | 0.002 | 0.935 | 0.065 | 0.997 | 0.003 |
| skouarn gat | dessert | France | Verger Conservatoire d’Arzano, Brittany | 0.952 | 0.048 | 0.997 | 0.003 | 0.995 | 0.005 | 0.998 | 0.002 |
| s2 sauvage moulin du roch | dessert | France | Verger Conservatoire d’Arzano, Brittany | 0.945 | 0.055 | 0.931 | 0.069 | 0.988 | 0.012 | 0.997 | 0.003 |
| s3 pommier hatif non identifié moulin du roch | dessert | France | Verger Conservatoire d’Arzano, Brittany | 0.536 | 0.464 | 0.999 | 0.001 | 0.995 | 0.005 | 0.996 | 0.004 |
| bacon ru | cider | France | Verger Conservatoire d’Arzano, Brittany | 0.972 | 0.028 | 0.999 | 0.001 | 0.997 | 0.003 | 0.998 | 0.002 |
| c’huello mverger moulin du roch | cider | France | Verger Conservatoire d’Arzano, Brittany | 0.966 | 0.034 | 0.999 | 0.001 | 0.996 | 0.004 | 0.998 | 0.002 |
| inconnue guy padan | cider | France | Verger Conservatoire d’Arzano, Brittany | 0.742 | 0.258 | 0.999 | 0.001 | 0.994 | 0.006 | 0.998 | 0.002 |
| dous coumoulen | cider | France | Verger Conservatoire d’Arzano, Brittany | 0.919 | 0.081 | 0.999 | 0.001 | 0.997 | 0.003 | 0.999 | 0.001 |
| kermerrien | cider | France | Verger Conservatoire d’Arzano, Brittany | 0.874 | 0.126 | 0.999 | 0.001 | 0.986 | 0.014 | 0.993 | 0.007 |
| dous bohars | dessert | France | Verger Conservatoire d’Arzano, Brittany | 0.96 | 0.04 | 0.998 | 0.002 | 0.997 | 0.003 | 0.998 | 0.002 |
| pomme orange | dessert | France | Verger Conservatoire d’Arzano, Brittany | 0.934 | 0.066 | 0.999 | 0.001 | 0.998 | 0.002 | 0.999 | 0.001 |
| poul poche | cider | France | Verger Conservatoire d’Arzano, Brittany | 0.902 | 0.098 | 0.999 | 0.001 | 0.997 | 0.003 | 0.999 | 0.001 |
| pomme cloc’h | cider | France | Verger Conservatoire d’Arzano, Brittany | 0.931 | 0.069 | 0.971 | 0.029 | 0.997 | 0.003 | 0.999 | 0.001 |
| commère | cider | France | Verger Conservatoire d’Arzano, Brittany | 0.876 | 0.124 | 0.998 | 0.002 | 0.997 | 0.003 | 0.999 | 0.001 |
| chuero bris | cider | France | Verger Conservatoire d’Arzano, Brittany | 0.873 | 0.127 | 0.997 | 0.003 | 0.995 | 0.005 | 0.994 | 0.006 |
| **Blanche de ste anne** | **dessert** | **France** | **Verger Conservatoire d’Arzano, Brittany** | **0.221** | **0.779** | **0.998** | **0.002** | **0.985** | **0.015** | **0.974** | **0.026** |
| tardive de la sarthe | cider | France | Verger Conservatoire d’Arzano, Brittany | 0.666 | 0.334 | 0.999 | 0.001 | 0.997 | 0.003 | 0.997 | 0.003 |
| bouteille | cider | France | Verger Conservatoire d’Arzano, Brittany | 0.927 | 0.073 | 0.977 | 0.023 | 0.993 | 0.007 | 0.997 | 0.003 |
| lost cam | cider | France | Verger Conservatoire d’Arzano, Brittany | 0.984 | 0.016 | 0.997 | 0.003 | 0.997 | 0.003 | 0.998 | 0.002 |
| fil rouge | dessert | France | Verger Conservatoire d’Arzano, Brittany | 0.975 | 0.025 | 0.998 | 0.002 | 0.995 | 0.005 | 0.997 | 0.003 |
| caot plom | cider | France | Verger Conservatoire d’Arzano, Brittany | 0.959 | 0.041 | 0.999 | 0.001 | 0.993 | 0.007 | 0.996 | 0.004 |
| dessus du paillé | dessert | France | Verger Conservatoire d’Arzano, Brittany | 0.801 | 0.199 | 0.999 | 0.001 | 0.996 | 0.004 | 0.996 | 0.004 |
| coat hir | cider | France | Verger Conservatoire d’Arzano, Brittany | 0.914 | 0.086 | 0.999 | 0.001 | 0.996 | 0.004 | 0.999 | 0.001 |
| fil jaune | dessert | France | Verger Conservatoire d’Arzano, Brittany | 0.845 | 0.155 | 0.998 | 0.002 | 0.997 | 0.003 | 0.998 | 0.002 |
| dous ribote | cider | France | Verger Conservatoire d’Arzano, Brittany | 0.89 | 0.11 | 0.999 | 0.001 | 0.976 | 0.024 | 0.994 | 0.006 |
| ste anne rouge | dessert | France | Verger Conservatoire d’Arzano, Brittany | 0.892 | 0.108 | 0.999 | 0.001 | 0.973 | 0.027 | 0.991 | 0.009 |
| dous kernao | cider | France | Verger Conservatoire d’Arzano, Brittany | 0.974 | 0.026 | 0.997 | 0.003 | 0.996 | 0.004 | 0.998 | 0.002 |
| justine | dessert | France | Verger Conservatoire d’Arzano, Brittany | 0.986 | 0.014 | 0.998 | 0.002 | 0.997 | 0.003 | 0.998 | 0.002 |
| dous glas | cider | France | Verger Conservatoire d’Arzano, Brittany | 0.97 | 0.03 | 0.999 | 0.001 | 0.985 | 0.015 | 0.997 | 0.003 |
| grise dieppoise | cider | France | Verger Conservatoire d’Arzano, Brittany | 0.954 | 0.046 | 0.999 | 0.001 | 0.995 | 0.005 | 0.996 | 0.004 |
| bienvenue | cider | France | Verger Conservatoire d’Arzano, Brittany | 0.88 | 0.12 | 0.999 | 0.001 | 0.997 | 0.003 | 0.999 | 0.001 |
| chailleux | dessert | France | Verger Conservatoire d’Arzano, Brittany | 0.895 | 0.105 | 0.998 | 0.002 | 0.995 | 0.005 | 0.997 | 0.003 |
| matheline | dessert | France | Verger Conservatoire d’Arzano, Brittany | 0.827 | 0.173 | 0.999 | 0.001 | 0.996 | 0.004 | 0.998 | 0.002 |
| doffage | cider | France | Verger Conservatoire d’Arzano, Brittany | 0.82 | 0.18 | 0.999 | 0.001 | 0.998 | 0.002 | 0.999 | 0.001 |
| tillet | dessert | France | Verger Conservatoire d’Arzano, Brittany | 0.907 | 0.093 | 0.998 | 0.002 | 0.998 | 0.002 | 0.998 | 0.002 |
| dessus du paillé | dessert | France | Verger Conservatoire d’Arzano, Brittany | 0.867 | 0.133 | 0.996 | 0.004 | 0.994 | 0.006 | 0.992 | 0.008 |
| carabille | dessert | France | Verger Conservatoire d’Arzano, Brittany | 0.625 | 0.375 | 0.999 | 0.001 | 0.994 | 0.006 | 0.993 | 0.007 |
| dous grise | cider | France | Verger Conservatoire d’Arzano, Brittany | 0.961 | 0.039 | 0.977 | 0.023 | 0.997 | 0.003 | 0.998 | 0.002 |
| reinette d’armorique | dessert | France | Verger Conservatoire d’Arzano, Brittany | 0.975 | 0.025 | 0.999 | 0.001 | 0.997 | 0.003 | 0.998 | 0.002 |
| 50 margueilt coz | cider | France | Verger Conservatoire d’Arzano, Brittany | 0.978 | 0.022 | 0.999 | 0.001 | 0.994 | 0.006 | 0.997 | 0.003 |
| dous gwen kerijan | cider | France | Verger Conservatoire d’Arzano, Brittany | 0.883 | 0.117 | 0.999 | 0.001 | 0.998 | 0.002 | 0.997 | 0.003 |
| bacon ru hatif | cider | France | Verger Conservatoire d’Arzano, Brittany | 0.693 | 0.307 | 0.999 | 0.001 | 0.917 | 0.083 | 0.979 | 0.021 |
| dous kervidan | cider | France | Verger Conservatoire d’Arzano, Brittany | 0.965 | 0.035 | 0.994 | 0.006 | 0.996 | 0.004 | 0.998 | 0.002 |
| kignet fri | cider | France | Verger Conservatoire d’Arzano, Brittany | 0.972 | 0.028 | 0.999 | 0.001 | 0.995 | 0.005 | 0.998 | 0.002 |
| reinette grise type de saintonge | dessert | France | Verger Conservatoire d’Arzano, Brittany | 0.898 | 0.102 | 0.999 | 0.001 | 0.996 | 0.004 | 0.997 | 0.003 |
| carabine | dessert | France | Verger Conservatoire d’Arzano, Brittany | 0.794 | 0.206 | 0.997 | 0.003 | 0.997 | 0.003 | 0.998 | 0.002 |
| bris canic | dessert | France | Verger Conservatoire d’Arzano, Brittany | 0.79 | 0.21 | 0.998 | 0.002 | 0.994 | 0.006 | 0.997 | 0.003 |
| dous moën | cider | France | Verger Conservatoire d’Arzano, Brittany | 0.636 | 0.364 | 0.998 | 0.002 | 0.995 | 0.005 | 0.996 | 0.004 |
| dous minotte | cider | France | Verger Conservatoire d’Arzano, Brittany | 0.659 | 0.341 | 0.994 | 0.006 | 0.972 | 0.028 | 0.991 | 0.009 |
| rené vert | dessert | France | Verger Conservatoire d’Arzano, Brittany | 0.987 | 0.013 | 0.973 | 0.027 | 0.996 | 0.004 | 0.999 | 0.001 |
| bacon bihan bris | cider | France | Verger Conservatoire d’Arzano, Brittany | 0.958 | 0.042 | 0.998 | 0.002 | 0.996 | 0.004 | 0.998 | 0.002 |
| fouesnen gwen | cider | France | Verger Conservatoire d’Arzano, Brittany | 0.724 | 0.276 | 0.998 | 0.002 | 0.995 | 0.005 | 0.997 | 0.003 |
| ru galand | dessert | France | Verger Conservatoire d’Arzano, Brittany | 0.964 | 0.036 | 0.999 | 0.001 | 0.995 | 0.005 | 0.998 | 0.002 |
| pen ognon | cider | France | Verger Conservatoire d’Arzano, Brittany | 0.984 | 0.016 | 0.999 | 0.001 | 0.997 | 0.003 | 0.998 | 0.002 |
| dous miliner | cider | France | Verger Conservatoire d’Arzano, Brittany | 0.983 | 0.017 | 0.997 | 0.003 | 0.996 | 0.004 | 0.997 | 0.003 |
| st quidic | dessert | France | Verger Conservatoire d’Arzano, Brittany | 0.695 | 0.305 | 0.999 | 0.001 | 0.995 | 0.005 | 0.943 | 0.057 |
| dous mad | cider | France | Verger Conservatoire d’Arzano, Brittany | 0.906 | 0.094 | 0.998 | 0.002 | 0.985 | 0.015 | 0.995 | 0.005 |
| couhen | cider | France | Verger Conservatoire d’Arzano, Brittany | 0.913 | 0.087 | 0.999 | 0.001 | 0.995 | 0.005 | 0.999 | 0.001 |
| dous bleud | cider | France | Verger Conservatoire d’Arzano, Brittany | 0.984 | 0.016 | 0.999 | 0.001 | 0.997 | 0.003 | 0.998 | 0.002 |
| eistek trink | dessert | France | Verger Conservatoire d’Arzano, Brittany | 0.914 | 0.086 | 0.998 | 0.002 | 0.992 | 0.008 | 0.997 | 0.003 |
| reinette de pont farcy | Pont | France | Verger Conservatoire d’Arzano, Brittany | 0.954 | 0.046 | 0.999 | 0.001 | 0.997 | 0.003 | 0.998 | 0.002 |
| mirebloaz | dessert | France | Verger Conservatoire d’Arzano, Brittany | 0.968 | 0.032 | 0.999 | 0.001 | 0.994 | 0.006 | 0.996 | 0.004 |
| dous mann | cider | France | Verger Conservatoire d’Arzano, Brittany | 0.924 | 0.076 | 0.998 | 0.002 | 0.997 | 0.003 | 0.998 | 0.002 |
| san adrian | cider | France | Verger Conservatoire d’Arzano, Brittany | 0.749 | 0.251 | 0.987 | 0.013 | 0.995 | 0.005 | 0.998 | 0.002 |
| pomme de vin moëlan | cider | France | Verger Conservatoire d’Arzano, Brittany | 0.966 | 0.034 | 0.998 | 0.002 | 0.993 | 0.007 | 0.996 | 0.004 |
| chuero jégo | dessert | France | Verger Conservatoire d’Arzano, Brittany | 0.804 | 0.196 | 0.998 | 0.002 | 0.99 | 0.01 | 0.997 | 0.003 |
| blanc duret | dessert | France | Verger Conservatoire d’Arzano, Brittany | 0.91 | 0.09 | 0.999 | 0.001 | 0.996 | 0.004 | 0.997 | 0.003 |
| médaille d’or | cider | France | Verger Conservatoire d’Arzano, Brittany | 0.969 | 0.031 | 0.998 | 0.002 | 0.996 | 0.004 | 0.998 | 0.002 |
| eistek lanester | cider | France | Verger Conservatoire d’Arzano, Brittany | 0.911 | 0.089 | 0.998 | 0.002 | 0.998 | 0.002 | 0.999 | 0.001 |
| **st jean** | **dessert** | **France** | **Verger Conservatoire d’Arzano, Brittany** | **0.266** | **0.734** | **0.999** | **0.001** | **0.977** | **0.023** | **0.968** | **0.032** |
| pomme sauvage auberge du cleuziou | dessert | France | Verger Conservatoire d’Arzano, Brittany | 0.771 | 0.229 | 0.999 | 0.001 | 0.96 | 0.04 | 0.962 | 0.038 |
| locard vert | dessert | France | Verger Conservatoire d’Arzano, Brittany | 0.963 | 0.037 | 0.999 | 0.001 | 0.993 | 0.007 | 0.994 | 0.006 |
| coat plom | cider | France | Verger Conservatoire d’Arzano, Brittany | 0.958 | 0.042 | 0.996 | 0.004 | 0.995 | 0.005 | 0.997 | 0.003 |
| sac’h biniou | cider | France | Verger Conservatoire d’Arzano, Brittany | 0.906 | 0.094 | 0.998 | 0.002 | 0.997 | 0.003 | 0.998 | 0.002 |
| dous veg bris | cider | France | Verger Conservatoire d’Arzano, Brittany | 0.815 | 0.185 | 0.999 | 0.001 | 0.997 | 0.003 | 0.998 | 0.002 |
| gwen penker diffon | cider | France | Verger Conservatoire d’Arzano, Brittany | 0.937 | 0.063 | 0.999 | 0.001 | 0.997 | 0.003 | 0.998 | 0.002 |
| avallou belein | cider | France | Verger Conservatoire d’Arzano, Brittany | 0.481 | 0.519 | 0.999 | 0.001 | 0.992 | 0.008 | 0.994 | 0.006 |
| chuero ru mentec | cider | France | Verger Conservatoire d’Arzano, Brittany | 0.847 | 0.153 | 0.999 | 0.001 | 0.998 | 0.002 | 0.997 | 0.003 |
| coat skorn | cider | France | Verger Conservatoire d’Arzano, Brittany | 0.983 | 0.017 | 0.998 | 0.002 | 0.997 | 0.003 | 0.998 | 0.002 |
| carter | cider | France | Verger Conservatoire d’Arzano, Brittany | 0.779 | 0.221 | 0.998 | 0.002 | 0.947 | 0.053 | 0.949 | 0.051 |
| joachim | dessert | France | Verger Conservatoire d’Arzano, Brittany | 0.943 | 0.057 | 0.999 | 0.001 | 0.997 | 0.003 | 0.998 | 0.002 |
| pont kor | cider | France | Verger Conservatoire d’Arzano, Brittany | 0.624 | 0.376 | 0.999 | 0.001 | 0.991 | 0.009 | 0.996 | 0.004 |
| greffen | dessert | France | Verger Conservatoire d’Arzano, Brittany | 0.97 | 0.03 | 0.999 | 0.001 | 0.996 | 0.004 | 0.998 | 0.002 |
| la galeuse | dessert | France | Verger Conservatoire d’Arzano, Brittany | 0.955 | 0.045 | 0.999 | 0.001 | 0.996 | 0.004 | 0.999 | 0.001 |
| bolomic | cider | France | Verger Conservatoire d’Arzano, Brittany | 0.925 | 0.075 | 0.998 | 0.002 | 0.997 | 0.003 | 0.998 | 0.002 |
| pen du | cider | France | Verger Conservatoire d’Arzano, Brittany | 0.471 | 0.529 | 0.997 | 0.003 | 0.997 | 0.003 | 0.995 | 0.005 |
| bienvenue | cider | France | Verger Conservatoire d’Arzano, Brittany | 0.984 | 0.016 | 0.999 | 0.001 | 0.998 | 0.002 | 0.999 | 0.001 |
| dous bloc’hic | cider | France | Verger Conservatoire d’Arzano, Brittany | 0.818 | 0.182 | 0.998 | 0.002 | 0.997 | 0.003 | 0.997 | 0.003 |
| ein shemer | dessert | Russia | USDA - PI 280401 | 0.681 | 0.319 | 0.998 | 0.002 | 0.995 | 0.005 | 0.987 | 0.013 |
| **antonovka 172670-b** | **cider** | **Russia** | **USDA - PI 589956** | **0.145** | **0.855** | **0.995** | **0.005** | **0.808** | **0.192** | **0.695** | **0.305** |
| korichnoe polosatoje | dessert | ? | USDA - PI 589491 | 0.61 | 0.39 | 0.999 | 0.001 | 0.831 | 0.169 | 0.792 | 0.208 |
| gravenstein washington red | dessert | ? | USDA - PI 588837 | 0.757 | 0.243 | 0.999 | 0.001 | 0.974 | 0.026 | 0.997 | 0.003 |
| **yellow transparent** | **dessert** | **Russia** | **USDA - PI 588859** | **0.217** | **0.783** | **0.999** | **0.001** | **0.961** | **0.039** | **0.957** | **0.043** |
| **antonovka kamenichka** | **dessert** | **Russia** | **USDA - PI 588995** | **0.211** | **0.789** | **0.999** | **0.001** | **0.837** | **0.163** | **0.949** | **0.051** |
| irish peach | dessert | Ireland | USDA - PI 104727 | 0.742 | 0.258 | 0.996 | 0.004 | 0.994 | 0.006 | 0.993 | 0.007 |
| koningszuur | dessert | Netherland | USDA - PI 188517 | 0.846 | 0.154 | 0.999 | 0.001 | 0.975 | 0.025 | 0.991 | 0.009 |
| poeltsamaa winter apple | dessert | ? | USDA - PI 383515 | 0.941 | 0.059 | 0.997 | 0.003 | 0.995 | 0.005 | 0.996 | 0.004 |
| ingol | dessert | ? | USDA - PI 589441 | 0.98 | 0.02 | 0.999 | 0.001 | 0.998 | 0.002 | 0.999 | 0.001 |
| **novosibirski sweet** | **dessert** | **Russie** | **USDA - PI 589478** | **0.263** | **0.737** | **0.999** | **0.001** | **0.976** | **0.024** | **0.977** | **0.023** |
| x a17 ap | cider | France | Abbaye de Beauport (France, Brittany) | 0.973 | 0.027 | 0.999 | 0.001 | 0.997 | 0.003 | 0.998 | 0.002 |
| x a18 ap | cider | France | Abbaye de Beauport (France, Brittany) | 0.983 | 0.017 | 0.999 | 0.001 | 0.995 | 0.005 | 0.998 | 0.002 |
| peau de chien a19 | cider | France | Abbaye de Beauport (France, Brittany) | 0.982 | 0.018 | 0.999 | 0.001 | 0.996 | 0.004 | 0.998 | 0.002 |
| x a22 ap | cider | France | Abbaye de Beauport (France, Brittany) | 0.986 | 0.014 | 0.998 | 0.002 | 0.998 | 0.002 | 0.999 | 0.001 |
| x a23 ap | cider | France | Abbaye de Beauport (France, Brittany) | 0.982 | 0.018 | 0.997 | 0.003 | 0.996 | 0.004 | 0.998 | 0.002 |
| x a28 ap | cider | France | Abbaye de Beauport (France, Brittany) | 0.983 | 0.017 | 0.998 | 0.002 | 0.997 | 0.003 | 0.999 | 0.001 |
| gros pigeonnet a29 | cider | France | Abbaye de Beauport (France, Brittany) | 0.983 | 0.017 | 0.999 | 0.001 | 0.998 | 0.002 | 0.999 | 0.001 |
| x a3 ap | cider | France | Abbaye de Beauport (France, Brittany) | 0.968 | 0.032 | 0.999 | 0.001 | 0.996 | 0.004 | 0.998 | 0.002 |
| x a4 ap | cider | France | Abbaye de Beauport (France, Brittany) | 0.958 | 0.042 | 0.998 | 0.002 | 0.995 | 0.005 | 0.998 | 0.002 |
| kemerrien a7 | cider | France | Abbaye de Beauport (France, Brittany) | 0.954 | 0.046 | 0.999 | 0.001 | 0.997 | 0.003 | 0.998 | 0.002 |
| kemerrien a8 | cider | France | Abbaye de Beauport (France, Brittany) | 0.937 | 0.063 | 0.998 | 0.002 | 0.996 | 0.004 | 0.998 | 0.002 |
| gros pigeonnet b1 | cider | France | Abbaye de Beauport (France, Brittany) | 0.975 | 0.025 | 0.997 | 0.003 | 0.998 | 0.002 | 0.999 | 0.001 |
| x b12 ap | cider | France | Abbaye de Beauport (France, Brittany) | 0.986 | 0.014 | 0.999 | 0.001 | 0.997 | 0.003 | 0.999 | 0.001 |
| poire b7 ap | cider | France | Abbaye de Beauport (France, Brittany) | 0.943 | 0.057 | 0.998 | 0.002 | 0.982 | 0.018 | 0.996 | 0.004 |
| reinette de pontrieux b8 | cider | France | Abbaye de Beauport (France, Brittany) | 0.985 | 0.015 | 0.995 | 0.005 | 0.997 | 0.003 | 0.998 | 0.002 |
| x b9r ap | cider | France | Abbaye de Beauport (France, Brittany) | 0.985 | 0.015 | 0.996 | 0.004 | 0.995 | 0.005 | 0.997 | 0.003 |
| x c123 ap | cider | France | Abbaye de Beauport (France, Brittany) | 0.917 | 0.083 | 0.995 | 0.005 | 0.997 | 0.003 | 0.998 | 0.002 |
| x c125 ap | cider | France | Abbaye de Beauport (France, Brittany) | 0.988 | 0.012 | 0.999 | 0.001 | 0.997 | 0.003 | 0.999 | 0.001 |
| x c134 ap | cider | France | Abbaye de Beauport (France, Brittany) | 0.977 | 0.023 | 0.997 | 0.003 | 0.998 | 0.002 | 0.999 | 0.001 |
| x c146 ap1 | cider | France | Abbaye de Beauport (France, Brittany) | 0.824 | 0.176 | 0.996 | 0.004 | 0.997 | 0.003 | 0.998 | 0.002 |
| x c146 ap2 | cider | France | Abbaye de Beauport (France, Brittany) | 0.856 | 0.144 | 0.998 | 0.002 | 0.996 | 0.004 | 0.998 | 0.002 |
| x c147 ap | cider | France | Abbaye de Beauport (France, Brittany) | 0.156 | 0.844 | 0.998 | 0.002 | 0.975 | 0.025 | 0.966 | 0.034 |
| x c148 ap | cider | France | Abbaye de Beauport (France, Brittany) | 0.982 | 0.018 | 0.998 | 0.002 | 0.998 | 0.002 | 0.999 | 0.001 |
| x c156 ap | cider | France | Abbaye de Beauport (France, Brittany) | 0.953 | 0.047 | 0.998 | 0.002 | 0.996 | 0.004 | 0.999 | 0.001 |
| tete de vache c160 | cider | France | Abbaye de Beauport (France, Brittany) | 0.485 | 0.515 | 0.998 | 0.002 | 0.991 | 0.009 | 0.968 | 0.032 |
| x d1 ap | cider | France | Abbaye de Beauport (France, Brittany) | 0.861 | 0.139 | 0.997 | 0.003 | 0.992 | 0.008 | 0.994 | 0.006 |
| gros pigeonnet d11 | cider | France | Abbaye de Beauport (France, Brittany) | 0.987 | 0.013 | 0.999 | 0.001 | 0.998 | 0.002 | 0.999 | 0.001 |
| gros pigeonnet d11 ap2 | cider | France | Abbaye de Beauport (France, Brittany) | 0.981 | 0.019 | 0.999 | 0.001 | 0.997 | 0.003 | 0.999 | 0.001 |
| x d3 ap | cider | France | Abbaye de Beauport (France, Brittany) | 0.794 | 0.206 | 0.994 | 0.006 | 0.965 | 0.035 | 0.998 | 0.002 |
| x d4 ap | cider | France | Abbaye de Beauport (France, Brittany) | 0.977 | 0.023 | 0.998 | 0.002 | 0.998 | 0.002 | 0.998 | 0.002 |
| x e1 ap | cider | France | Abbaye de Beauport (France, Brittany) | 0.986 | 0.014 | 0.999 | 0.001 | 0.997 | 0.003 | 0.999 | 0.001 |
| x e4 ap | cider | France | Abbaye de Beauport (France, Brittany) | 0.59 | 0.41 | 0.999 | 0.001 | 0.966 | 0.034 | 0.981 | 0.019 |
| x f2 ap | cider | France | Abbaye de Beauport (France, Brittany) | 0.953 | 0.047 | 0.985 | 0.015 | 0.996 | 0.004 | 0.997 | 0.003 |
| x f3 ap | cider | France | Abbaye de Beauport (France, Brittany) | 0.949 | 0.051 | 0.998 | 0.002 | 0.996 | 0.004 | 0.997 | 0.003 |
| x f5 ap | cider | France | Abbaye de Beauport (France, Brittany) | 0.975 | 0.025 | 0.986 | 0.014 | 0.994 | 0.006 | 0.997 | 0.003 |
| x rear ap | cider | France | Abbaye de Beauport (France, Brittany) | 0.984 | 0.016 | 0.999 | 0.001 | 0.997 | 0.003 | 0.998 | 0.002 |
| x repi ap | cider | France | Abbaye de Beauport (France, Brittany) | 0.988 | 0.012 | 0.998 | 0.002 | 0.998 | 0.002 | 0.999 | 0.001 |
| x ropl ap | cider | France | Abbaye de Beauport (France, Brittany) | 0.956 | 0.044 | 0.974 | 0.026 | 0.996 | 0.004 | 0.998 | 0.002 |
| M8 | dessert | Armenia | Field sampling | 0.958 | 0.042 | 0.931 | 0.069 | 0.913 | 0.087 | 0.963 | 0.037 |
| Shagarkeni | dessert | Armenia | Field sampling | 0.838 | 0.162 | 0.938 | 0,062 | 0.909 | 0.091 | 0.768 | 0.232 |
| fenouillet gris | dessert | France | INRA | 0.985 | 0.015 | 0.995 | 0.005 | 0.997 | 0.003 | 0.998 | 0.002 |
| belle fleur jaune | dessert | USA | INRA | 0.985 | 0.015 | 0.997 | 0.003 | 0.998 | 0.002 | 0.998 | 0.002 |
| calville blanc d'hiver | dessert | Switzerland | INRA | 0.987 | 0.013 | 0.972 | 0.028 | 0.998 | 0.002 | 0.999 | 0.001 |
| gros api | dessert | France | INRA | 0.587 | 0.413 | 0.999 | 0.001 | 0.948 | 0.052 | 0.992 | 0.008 |
| reinette de cuzy | dessert | France | INRA | 0.841 | 0.159 | 0.999 | 0.001 | 0.997 | 0.003 | 0.998 | 0.002 |
| bec d'oie | dessert | France | INRA | 0.973 | 0.027 | 0.993 | 0.007 | 0.997 | 0.003 | 0.998 | 0.002 |
| amere de berthecourt | cider | France | INRA | 0.904 | 0.096 | 0.999 | 0.001 | 0.996 | 0.004 | 0.995 | 0.005 |
| armagnac | cider | France | INRA | 0.963 | 0.037 | 0.662 | 0.338 | 0.996 | 0.004 | 0.999 | 0.001 |
| bassard | cider | France | INRA | 0.979 | 0.021 | 0.998 | 0.002 | 0.998 | 0.002 | 0.999 | 0.001 |
| bedange rouge | cider | France | INRA | 0.977 | 0.023 | 0.998 | 0.002 | 0.997 | 0.003 | 0.998 | 0.002 |
| binet blanc | cider | France | INRA | 0.887 | 0.113 | 0.998 | 0.002 | 0.99 | 0.01 | 0.993 | 0.007 |
| binet gris | cider | France | INRA | 0.971 | 0.029 | 0.999 | 0.001 | 0.998 | 0.002 | 0.999 | 0.001 |
| blanc mollet | cider | France | INRA | 0.882 | 0.118 | 0.998 | 0.002 | 0.996 | 0.004 | 0.999 | 0.001 |
| cahoua | cider | France | INRA | 0.941 | 0.059 | 0.999 | 0.001 | 0.994 | 0.006 | 0.998 | 0.002 |
| michelin | cider | France | INRA | 0.983 | 0.017 | 0.999 | 0.001 | 0.996 | 0.004 | 0.998 | 0.002 |
| moulin a vent de l'eure | cider | France | INRA | 0.797 | 0.203 | 0.998 | 0.002 | 0.996 | 0.004 | 0.998 | 0.002 |
| moulin a vent du calvados | cider | France | INRA | 0.983 | 0.017 | 0.999 | 0.001 | 0.995 | 0.005 | 0.998 | 0.002 |
| petit gilet rouge de janze | cider | France | INRA | 0.883 | 0.117 | 0.998 | 0.002 | 0.983 | 0.017 | 0.987 | 0.013 |
| petite sorte du parc dufour | cider | France | INRA | 0.954 | 0.046 | 0.998 | 0.002 | 0.997 | 0.003 | 0.997 | 0.003 |
| reinette d'armorique | cider | France | INRA | 0.979 | 0.021 | 0.999 | 0.001 | 0.997 | 0.003 | 0.998 | 0.002 |
| stang ru | cider | France | INRA | 0.634 | 0.366 | 0.999 | 0.001 | 0.995 | 0.005 | 0.992 | 0.008 |
| cartigny | cider | France | INRA | 0.91 | 0.09 | 0.999 | 0.001 | 0.998 | 0.002 | 0.998 | 0.002 |
| mettais | cider | France | INRA | 0.692 | 0.308 | 0.999 | 0.001 | 0.994 | 0.006 | 0.998 | 0.002 |
| sebin blanc | cider | France | INRA | 0.983 | 0.017 | 0.999 | 0.001 | 0.997 | 0.003 | 0.998 | 0.002 |
| clos renaux | cider | France | INRA | 0.965 | 0.035 | 0.997 | 0.003 | 0.973 | 0.027 | 0.997 | 0.003 |
| binet violet | cider | France | INRA | 0.796 | 0.204 | 0.999 | 0.001 | 0.992 | 0.008 | 0.997 | 0.003 |
| clara | cider | Spain | INRA | 0.939 | 0.061 | 0.998 | 0.002 | 0.995 | 0.005 | 0.998 | 0.002 |
| bisquet | cider | France | INRA | 0.985 | 0.015 | 0.997 | 0.003 | 0.995 | 0.005 | 0.998 | 0.002 |
| egyptia | cider | France | INRA | 0.941 | 0.059 | 0.997 | 0.003 | 0.997 | 0.003 | 0.999 | 0.001 |
| saint martin | cider | France | INRA | 0.986 | 0.014 | 0.995 | 0.005 | 0.997 | 0.003 | 0.998 | 0.002 |
| jeanne renard | cider | France | INRA | 0.884 | 0.116 | 0.999 | 0.001 | 0.997 | 0.003 | 0.999 | 0.001 |
| judin | cider | France | INRA | 0.976 | 0.024 | 0.999 | 0.001 | 0.997 | 0.003 | 0.999 | 0.001 |
| api etoile | dessert | France | INRA | 0.497 | 0.503 | 0.997 | 0.003 | 0.777 | 0.223 | 0.979 | 0.021 |
| chuero ru | cider | France | INRA | 0.945 | 0.055 | 0.997 | 0.003 | 0.993 | 0.007 | 0.996 | 0.004 |
| avrolles | cider | France | INRA | 0.959 | 0.041 | 0.999 | 0.001 | 0.997 | 0.003 | 0.998 | 0.002 |
| doux veret de carrouges | cider | France | INRA | 0.852 | 0.148 | 0.999 | 0.001 | 0.998 | 0.002 | 0.999 | 0.001 |
| blanc sur | cider | France | INRA | 0.988 | 0.012 | 0.997 | 0.003 | 0.997 | 0.003 | 0.999 | 0.001 |
| petit rouget de dol | cider | France | INRA | 0.989 | 0.011 | 0.997 | 0.003 | 0.997 | 0.003 | 0.999 | 0.001 |
| chevalier jaune | cider | France | INRA | 0.869 | 0.131 | 0.999 | 0.001 | 0.996 | 0.004 | 0.999 | 0.001 |
| petit jaune (loire alantique) | cider | France | INRA | 0.874 | 0.126 | 0.997 | 0.003 | 0.996 | 0.004 | 0.999 | 0.001 |
| guillevic | cider | France | INRA | 0.98 | 0.02 | 0.999 | 0.001 | 0.996 | 0.004 | 0.998 | 0.002 |
| rene martin | cider | France | INRA | 0.955 | 0.045 | 0.998 | 0.002 | 0.997 | 0.003 | 0.999 | 0.001 |
| jaune de vitre | cider | France | INRA | 0.98 | 0.02 | 0.999 | 0.001 | 0.997 | 0.003 | 0.999 | 0.001 |
| binet rouge | cider | France | INRA | 0.977 | 0.023 | 0.999 | 0.001 | 0.994 | 0.006 | 0.998 | 0.002 |
| doux joseph | cider | France | INRA | 0.984 | 0.016 | 0.999 | 0.001 | 0.998 | 0.002 | 0.999 | 0.001 |
| crollon | cider | France | INRA | 0.976 | 0.024 | 0.998 | 0.002 | 0.997 | 0.003 | 0.999 | 0.001 |
| mariennet | cider | France | INRA | 0.981 | 0.019 | 0.97 | 0.03 | 0.997 | 0.003 | 0.999 | 0.001 |
| petit amer | cider | France | INRA | 0.98 | 0.02 | 0.739 | 0.261 | 0.995 | 0.005 | 0.998 | 0.002 |
| amere saint jacques | cider | France | INRA | 0.87 | 0.13 | 0.999 | 0.001 | 0.996 | 0.004 | 0.998 | 0.002 |
| doux eveque briz | cider | France | INRA | 0.823 | 0.177 | 0.999 | 0.001 | 0.997 | 0.003 | 0.998 | 0.002 |
| chuero ru bihan | cider | France | INRA | 0.956 | 0.044 | 0.999 | 0.001 | 0.93 | 0.07 | 0.987 | 0.013 |
| chuero ru mod koz | cider | France | INRA | 0.978 | 0.022 | 0.884 | 0.116 | 0.998 | 0.002 | 0.999 | 0.001 |
| treujenn hir | cider | France | INRA | 0.949 | 0.051 | 0.997 | 0.003 | 0.995 | 0.005 | 0.996 | 0.004 |
| prat yeot | cider | France | INRA | 0.947 | 0.053 | 0.999 | 0.001 | 0.998 | 0.002 | 0.998 | 0.002 |
| avalou belein | cider | France | INRA | 0.759 | 0.241 | 0.999 | 0.001 | 0.996 | 0.004 | 0.997 | 0.003 |
| doux au gobet | cider | France | INRA | 0.637 | 0.363 | 0.999 | 0.001 | 0.993 | 0.007 | 0.997 | 0.003 |
| cossa | cider | France | INRA | 0.947 | 0.053 | 0.999 | 0.001 | 0.979 | 0.021 | 0.996 | 0.004 |
| doux corier | cider | France | INRA | 0.964 | 0.036 | 0.999 | 0.001 | 0.997 | 0.003 | 0.999 | 0.001 |
| marseigna | cider | France | INRA | 0.915 | 0.085 | 0.996 | 0.004 | 0.994 | 0.006 | 0.997 | 0.003 |
| patte de loup | dessert | France | INRA | 0.981 | 0.019 | 0.999 | 0.001 | 0.997 | 0.003 | 0.999 | 0.001 |
| colapuis | dessert | Ukraine | INRA | 0.718 | 0.282 | 0.998 | 0.002 | 0.989 | 0.011 | 0.996 | 0.004 |
| non pareil | dessert | France | INRA | 0.987 | 0.013 | 0.999 | 0.001 | 0.997 | 0.003 | 0.998 | 0.002 |

? : Unknown origin
