# Supplementary material for: New Insight into the History of Domesticated Apple: Secondary Contribution of the European Wild Apple to the Genome of Cultivated Varieties
Source: PLoS Genet. 2012 May 10;8(5):e1002703. doi: 10.1371/journal.pgen.1002703 (PMC3349737; doi:10.1371/journal.pgen.1002703)
Supplement: Table S3 — Membership coefficients inferred from the STRUCTURE analysis for M. baccata individuals. (DOC) [file pgen.1002703.s006.doc]

Table S3. Membership coefficients inferred from the STRUCTURE analysis for *Malus baccata* individuals. Names in bold correspond to *M. baccata* introgressed by *M. domestica.*

| Origin/cultivar name* | | | *M. baccata* | *M. domestica* |  |
| --- | --- | --- | --- | --- | --- |
| Russia | | | 0.999 | 0.001 |  |
| Russia | | | 0.996 | 0.004 |  |
| Russia | | | 0.898 | 0.102 |  |
| Russia | | | 0.999 | 0.001 |  |
| Russia | | | 0.999 | 0.001 |  |
| Russia | | | 0.951 | 0.049 |  |
| Russia | | | 0.999 | 0.001 |  |
| Russia | | | 0.997 | 0.003 |  |
| Russia | | | 0.999 | 0.001 |  |
| Russia | | | 0.999 | 0.001 |  |
| Russia | | | 0.999 | 0.001 |  |
| Russia | | | 0.999 | 0.001 |  |
| Russia | | | 0.999 | 0.001 |  |
| Russia | | | 0.886 | 0.114 |  |
| Russia | | | 0.998 | 0.002 |  |
| Russia | | | 0.999 | 0.001 |  |
| Russia | | | 0.998 | 0.002 |  |
| Russia | | | 0.999 | 0.001 |  |
| Russia | | | 0.999 | 0.001 |  |
| Russia | | | 0.988 | 0.012 |  |
| Russia | | | 0.997 | 0.003 |  |
| Russia | | | 0.999 | 0.001 |  |
| Russia | | | 0.648 | 0.352 |  |
| Russia | | | 0.999 | 0.001 |  |
| Russia | | | 0.999 | 0.001 |  |
| Russia | | | 0.971 | 0.029 |  |
| Russia | | | 0.999 | 0.001 |  |
| Russia | | | 0.999 | 0.001 |  |
| Russia | | | 0.998 | 0.002 |  |
| Russia | | | 0.999 | 0.001 |  |
| Russia | | | 0.997 | 0.003 |  |
| Russia | | | 0.725 | 0.275 |  |
| Russia | | | 0.999 | 0.001 |  |
| Russia | | | 0.978 | 0.022 |  |
| Russia | | | 0.995 | 0.005 |  |
| **Russia** | | | **0.667** | **0.333** |  |
| Romania | | | 0.973 | 0.027 |  |
| **Hungary** | | | **0.19** | **0.81** |  |
| unknown (EMR^1^) | | | 0.989 | 0.011 |  |
| ***flexilis (*EMR^1^*)*** | | | **0.653** | **0.347** |  |
| *gracilis (*EMR^1^*)* | | | 0.155 | 0.845 |  |
| *jackii (*EMR^1^*)* | | | 0.702 | 0.298 |  |
| ***mandshurica (USDA-ARS^2^. gmal35)*** | | | **0.569** | **0.431** |  |
| *rockii (USDA-ARS^2^. gmal423)* | | | 0.985 | 0.015 |  |
| ***flexilis (USDA-ARS^2^. gmal1605)*** | | | **0.674** | **0.326** |  |
| unknown (*USDA-ARS^2^*. gmal1617) | | | 0.998 | 0.002 |  |
| *jackii (USDA-ARS^2^. gmal2460)* | | | 0.998 | 0.002 |  |
| ***Hansen's (USDA-ARS^2^. gmal2477)*** | | | **0.464** | **0.536** |  |
|  | * Samples whose origin is indicated as “Russia” were effectively collected in Russia. Samples from apple collections from Romania or Hungary are actually of unknown geographic origin. For all other samples, variety names are given, with germplasm repository names in parentheses. | | | | |
|  | EMR^1^ | East Malling Researh, Kent, UK | | | |
|  | *USDA-ARS^2^* | United States Departement of Agriculture - Agricultural Research Service, Cornell | | | |

University, USA
