# Supplementary material for: New Insight into the History of Domesticated Apple: Secondary Contribution of the European Wild Apple to the Genome of Cultivated Varieties
Source: PLoS Genet. 2012 May 10;8(5):e1002703. doi: 10.1371/journal.pgen.1002703 (PMC3349737; doi:10.1371/journal.pgen.1002703)
Supplement: Table S4 — Prior distributions used in approximate Bayesian computations. Prior distributions are uniform between lower and upper bound. Parameters are introduced in Figure 4 and Table 5. Species names are abbreviated. (DOC) [file pgen.1002703.s007.doc]

**Table S4.** Prior distributions used in approximate Bayesian computations. Prior distributions are uniform between lower and upper bound. Parameters are introduced in Figure 4 and Table 5. Species names are abbreviated.

| **Parameter** | **Lower bound** | **Upper bound** |
| --- | --- | --- |
| *N1* (*M. dom*) | 1 | 6,000 |
| *N2* (*M. ori*) | 1,000 | 70,000 |
| *N3* (*M. siev*) | 1,000 | 20,000 |
| *N4* (*M. sylv*) | 1,000 | 50,000 |
| *T1* (*M. siev* - *M.sylv*) | 615 | 50,000 |
| *T2* (*M. siev* - *M. ori*) | 615 | 7,000 |
| *T3* (*M. siev* - *M. dom*) | 614 | 3,500 |
| *r1* (introgr. by *M. dom* into *M. siev*) | 0.001 | 0.700 |
| *r2* (introgr. by *M. sylv* into *M. dom*) | 0.001 | 0.400 |
| *r3* (introgr. by *M. dom* into *M. sylv*) | 0.001 | 0.700 |
| *r4* (introgr. by *M. dom* into *M. ori*) | 0.001 | 0.400 |
| *r5* (introgr. by *M. ori* into *M. dom*) | 0.001 | 0.700 |
| *μ* | 10^-4^ | 10^-3^ |
| *p* | 0.10 | 0.30 |
| *μSNI* | 10^-8^ | 10^-4^ |
