# Supplementary material for: New Insight into the History of Domesticated Apple: Secondary Contribution of the European Wild Apple to the Genome of Cultivated Varieties
Source: PLoS Genet. 2012 May 10;8(5):e1002703. doi: 10.1371/journal.pgen.1002703 (PMC3349737; doi:10.1371/journal.pgen.1002703)
Supplement: Table S5 — Relative posterior probabilities (p) for the four historical models compared using approximate Bayesian computations. Models are described in Figure 4. CI2.5 and CI97.5 are boundaries of the 95% confidence intervals. (A) Analyses on a pruned dataset with misclassified wild individuals and individuals with a recent admixed ancestry removed. (B) Analyses on the full dataset, assuming that admixture between ancestral M. domestica and M. sylvestris was more recent (67 generations–500 ybp) than in original analyses (200 generations–1,500 ybp). (DOC) [file pgen.1002703.s008.doc]

**Table S5.** Relative posterior probabilities (*p*) for the four historical models compared using approximate Bayesian computations. Models are described in Figure 4. CI2.5 and CI97.5 are boundaries of the 95% confidence intervals. (A) Analyses on a pruned dataset with misclassified wild individuals and individuals with a recent admixed ancestry removed. (B) Analyses on the full dataset, assuming that admixture between ancestral *M. domestica* and *M. sylvestris* was more recent (67 generations, 500 ybp) than in original analyses (200 generations, 1500 ybp).

| **Treatment/Model** | | ***p*** | **CI2.5** | **CI97.5** |
| --- | --- | --- | --- | --- |
| A |  |  |  |  |
|  | *a* | 0.0008 | 0.0005 | 0.0011 |
|  | *b* | 0.5135 | 0.4778 | 0.5492 |
|  | *c* | 0.4857 | 0.4500 | 0.5214 |
|  | *d* | 0.0000 | 0.0000 | 0.0000 |
| B |  |  |  |  |
|  | *a* | 0.0360 | 0.0240 | 0.0480 |
|  | *b* | 0.4154 | 0.3765 | 0.4543 |
|  | *c* | 0.5486 | 0.5089 | 0.5882 |
|  | *d* | 0.0000 | 0.0000 | 0.0000 |
