# Supplementary material for: New Insight into the History of Domesticated Apple: Secondary Contribution of the European Wild Apple to the Genome of Cultivated Varieties
Source: PLoS Genet. 2012 May 10;8(5):e1002703. doi: 10.1371/journal.pgen.1002703 (PMC3349737; doi:10.1371/journal.pgen.1002703)
Supplement: Table S6 — Demographic and mutation parameters estimated using approximate Bayesian computation for model c. Posterior distributions are summarized as the mode and boundaries of the 95% credibility intervals (CI2.5 and CI97.5). Demographic parameters are introduced in Figure 4 (note that admixture times are fixed in these analyses). Composite parameters scaled by the mutation rate are also shown. The mutation parameters are μ (mean mutation rate), p (mean value of the geometric distribution parameter that governs the number of repeated motifs that increase or decrease the length of the locus during mutation events), μSNI (mean single nucleotide indel mutation rate). Species names are abbreviated. (A) Analyses on a pruned dataset with misclassified wild individuals and individuals with a recent admixed ancestry removed. (B) Analyses on the full dataset, assuming that admixture between ancestral M. domestica and M. sylvestris was more recent (67 generations–500 ybp) than in original analyses (200 generations–1,500 ybp). (DOC) [file pgen.1002703.s009.doc]

**Table S6.** Demographic and mutation parameter estimated using approximate Bayesian computation for model *c*. Posterior distributions are summarized as the mode and boundaries of the 95% credibility intervals (CI2.5 and CI97.5). Demographic parameters are introduced in Figure 4 (note that admixture times are fixed in these analyses). Composite parameters scaled by the mutation rate are also shown. The mutation parameters are *μ* (mean mutation rate), *p* (mean value of the geometric distribution parameter that governs the number of repeated motifs that increase or decrease the length of the locus during mutation events), *μSNI* (mean single nucleotide indel mutation rate). Species names are abbreviated. (A) Analyses on a pruned dataset with misclassified wild individuals and individuals with a recent admixed ancestry removed. (B) Analyses on the full dataset, assuming that admixture between ancestral *M. domestica* and *M. sylvestris* was more recent (67 generations – 500 ybp) than in original analyses (200 generations – 1500 ybp).

| **Parameter** | | **Treatment A** | | |  | **Treatment B** | | |
| --- | --- | --- | --- | --- | --- | --- | --- | --- |
|  |  | **Mode** | **CI2.5** | **CI97.5** |  | **Mode** | **CI2.5** | **CI97.5** |
|  | *N1* (*M. dom*) | 1470 | 844 | 4250 |  | 1410 | 860 | 5090 |
|  | *N2* (*M. ori*) | 40200 | 17000 | 67700 |  | 27000 | 10900 | 63500 |
|  | *N3* (*M. siev*) | 4800 | 2480 | 12300 |  | 14200 | 6710 | 19400 |
|  | *N4* (*M. sylv*) | 33700 | 16300 | 48700 |  | 32900 | 16000 | 48400 |
|  | *T1* (*M. siev* - *M.sylv*) | 9270 | 4900 | 45000 |  | 11600 | 5060 | 44500 |
|  | *T2* (*M. siev* - *M. ori*) | 2340 | 1100 | 6260 |  | 2480 | 1220 | 6070 |
|  | *T3* (*M. siev* - *M. dom*) | 1690 | 746 | 3400 |  | 1560 | 758 | 3300 |
|  | *r2* (introgr. by *M. sylv* into *M. dom*) | 0.65 | 0.50 | 0.69 |  | 0.63 | 0.51 | 0.68 |
|  | *μ* | 2.2.10^-4^ | 1.1.10^-4^ | 7.8.10^-4^ |  | 1.7.10^-4^ | 1.1.10^-4^ | 6.4.10^-4^ |
|  | *p* | 0.3 | 0.1 | 0.3 |  | 0.3 | 0.1 | 0.3 |
|  | *μSNI* | 3.0.10^-8^ | 4.0.10^-8^ | 5.2.10^-5^ |  | 4.0.10^-8^ | 7.0.10^-8^ | 6.1.10^-5^ |
|  | *θ1* (=4*N1μ*) | 0.43 | 0.21 | 1.54 |  | 0.36 | 0.19 | 0.17 |
|  | *θ2* (=4*N2μ*) | 10.8 | 4.8 | 34.2 |  | 4.6 | 2.8 | 22.0 |
|  | *θ3* (=4*N3μ*) | 1.5 | 0.9 | 3.8 |  | 2.7 | 1.8 | 7.0 |
|  | *θ4* (=4*N4μ*) | 8.3 | 4.8 | 22.0 |  | 6.7 | 4.2 | 17.9 |
|  | *τ1* (=*μT1*) | 3.2 | 1.4 | 18.8 |  | 2.6 | 1.2 | 15.4 |
|  | *τ2* (=*μT2*) | 0.6 | 0.3 | 2.6 |  | 0.5 | 0.3 | 2.2 |
|  | *τ3* (=*μT3*) | 0.5 | 0.2 | 1.7 |  | 0.3 | 0.2 | 1.3 |
