# Supplementary material for: New Insight into the History of Domesticated Apple: Secondary Contribution of the European Wild Apple to the Genome of Cultivated Varieties
Source: PLoS Genet. 2012 May 10;8(5):e1002703. doi: 10.1371/journal.pgen.1002703 (PMC3349737; doi:10.1371/journal.pgen.1002703)
Supplement: Table S7 — Model checking based on comparisons of test quantities between observed data and 100 pseudo-observed datasets generated using parameter values drawn from posterior distributions. (A) Analyses on the full dataset, (B) Analyses on a pruned dataset with misclassified wild individuals and individuals with a recent admixed ancestry removed. (C) Analyses on the full dataset, assuming that admixture between ancestral Malus domestica and M. sylvestris was more recent (67 generations–500 ybp) than in (A). (DOC) [file pgen.1002703.s010.doc]

**Table S7.** Model checking based on comparisons of test quantities between observed data and 100 pseudo-observed datasets generated using parameter values drawn from posterior distributions. (A) Analyses on the full dataset, (B) Analyses on a pruned dataset with misclassified wild individuals and individuals with a recent admixed ancestry removed. (C) Analyses on the full dataset, assuming that admixture between ancestral *Malus domestica* and *M. sylvestris* was more recent (67 generations, 500 ybp) than in (A).

| Test quantity | Tail-area probability | | | | | | | | | | | | | | |
| --- | --- | --- | --- | --- | --- | --- | --- | --- | --- | --- | --- | --- | --- | --- | --- |
|  | Treatment A | | | | | Treatment B | | | | | Treatment C | | | | |
|  | Observed value | *Model a* | *Model b* | *Model c* | *Model d* | Observed value | *Model a* | *Model b* | *Model c* | *Model d* | Observed value | *Model a* | *Model b* | *Model c* | *Model d* |
| *VAR1* | 32.05 | 0.34 | 0.36 | 0.33 | 0.62 | 29.74 | 0.25 | 0.31 | 0.24 | 0.86 | 32.05 | 0.32 | 0.39 | 0.38 | 0.77 |
| *VAR2* | 19.18 | 0.51 | 0.25 | 0.16 | 0.2 | 17.58 | 0.68 | 0.45 | 0.49 | 0.45 | 19.18 | 0.42 | 0.23 | 0.18 | 0.23 |
| *VAR3* | 21.17 | 0.6 | 0.35 | 0.26 | 0.37 | 15.15 | 0.69 | 0.52 | 0.57 | 0.62 | 21.17 | 0.59 | 0.39 | 0.29 | 0.45 |
| *VAR4* | 32.02 | 0.38 | 0.39 | 0.38 | 0.35 | 30.63 | 0.25 | 0.33 | 0.29 | 0.29 | 32.02 | 0.345 | 0.43 | 0.35 | 0.49 |
| *N2P12* | 23.57 | 0.25 | 0.36 | 0.275 | 0.605 | 21.00 | 0.27 | 0.355 | 0.41 | 0.68 | 23.57 | 0.27 | 0.265 | 0.245 | 0.52 |
| *N2P13* | 20.64 | 0.245 | 0.19 | 0.17 | 0.61 | 16.64 | 0.23 | 0.265 | 0.32 | 0.725 | 20.64 | 0.24 | 0.21 | 0.185 | 0.59 |
| *N2P14* | 21.79 | 0.33 | 0.45 | 0.415 | 0.36 | 19.64 | 0.3 | 0.27 | 0.33 | 0.26 | 21.79 | 0.415 | 0.4 | 0.46 | 0.34 |
| *N2P23* | 21.00 | 0.295 | 0.46 | 0.32 | 0.45 | 17.36 | 0.38 | 0.375 | 0.41 | 0.38 | 21.00 | 0.33 | 0.46 | 0.39 | 0.29 |
| *N2P24* | 24.14 | 0.29 | 0.34 | 0.285 | 0.45 | 23.86 | 0.28 | 0.33 | 0.38 | 0.395 | 24.14 | 0.33 | 0.3 | 0.33 | 0.395 |
| *N2P34* | 21.86 | 0.355 | 0.29 | 0.28 | 0.405 | 20.50 | 0.315 | 0.28 | 0.365 | 0.39 | 21.86 | 0.39 | 0.27 | 0.275 | 0.41 |
| *H2P12* | 0.871 | 0.17 | 0.115 | 0.135 | 0.445 | 0.860 | 0.285 | 0.26 | 0.305 | 0.5 | 0.871 | 0.2 | 0.175 | 0.175 | 0.345 |
| *H2P13* | 0.866 | 0.25 | 0.09 | 0.165 | 0.58 | 0.852 | 0.315 | 0.35 | 0.395 | 0.755 | 0.866 | 0.2 | 0.17 | 0.23 | 0.57 |
| *H2P14* | 0.862 | 0.135 | 0.1 | 0.145 | 0.585 | 0.8541 | 0.175 | 0.2 | 0.285 | 0.67 | 0.862 | 0.1 | 0.15 | 0.215 | 0.53 |
| *H2P23* | 0.832 | 0.245 | 0.115 | 0.155 | 0.12 | 0.807 | 0.3 | 0.245 | 0.275 | 0.125 | 0.832 | 0.27 | 0.17 | 0.135 | 0.09 |
| *H2P24* | 0.852 | 0.175 | 0.09 | 0.13 | 0.11 | 0.843 | 0.235 | 0.25 | 0.24 | 0.135 | 0.852 | 0.19 | 0.155 | 0.13 | 0.09 |
| *H2P34* | 0.847 | 0.38 | 0.175 | 0.23 | 0.23 | 0.826 | 0.39 | 0.345 | 0.325 | 0.35 | 0.847 | 0.435 | 0.195 | 0.155 | 0.255 |
| *DAS12* | 0.091 | 0.69 | 0.795 | 0.77 | 0.45 | 0.089 | 0.475 | 0.645 | 0.54 | 0.325 | 0.091 | 0.585 | 0.72 | 0.795 | 0.535 |
| *DAS13* | 0.098 | 0.67 | 0.85 | 0.795 | 0.415 | 0.089 | 0.535 | 0.53 | 0.445 | 0.18 | 0.098 | 0.62 | 0.775 | 0.75 | 0.435 |
| *DAS14* | 0.095 | 0.7 | 0.81 | 0.74 | 0.965* | 0.084 | 0.73 | 0.725 | 0.72 | 0.975* | 0.095 | 0.575 | 0.765 | 0.77 | 0.925 |
| *DAS23* | 0.148 | 0.765 | 0.85 | 0.84 | 0.89 | 0.174 | 0.68 | 0.78 | 0.75 | 0.915 | 0.148 | 0.76 | 0.835 | 0.84 | 0.94 |
| *DAS24* | 0.057 | 0.52 | 0.67 | 0.655 | 0.545 | 0.050 | 0.475 | 0.69 | 0.64 | 0.635 | 0.057 | 0.515 | 0.675 | 0.655 | 0.61 |
| *DAS34* | 0.063 | 0.535 | 0.83 | 0.75 | 0.685 | 0.051 | 0.515 | 0.705 | 0.62 | 0.665 | 0.063 | 0.555 | 0.755 | 0.745 | 0.7 |

*1*: *M. domestica*; 2: *M. orientalis*; 3: *M. sieversii*; 4: *M. sylvestris*. Tail-area probability was computed for each test quantities (*tq*) as *p* and 1 – *p* for *p* ≤ 0.5 and > 0.5, respectively, with *p*= *Prob[tq(simulated)<tq(observed)]* (Cornuet et al. 2010). *VARi* = mean allelic size variance in population *i*, *N2Pij* = mean number of alleles in populations *i* and *j*, *H2Pij* = mean gene diversity in populations *i* and *j*, *DASij* = proportion of shared alleles between populations *i* and *j*. * *p*>0.95.
