# Supplementary material for: New Insight into the History of Domesticated Apple: Secondary Contribution of the European Wild Apple to the Genome of Cultivated Varieties
Source: PLoS Genet. 2012 May 10;8(5):e1002703. doi: 10.1371/journal.pgen.1002703 (PMC3349737; doi:10.1371/journal.pgen.1002703)
Supplement: Table S8 — Genetic differentiation (FST) between cultivars of different geographic origins (N = 266). Cultivars of unknown origin have been removed. (DOC) [file pgen.1002703.s011.doc]

Table S8: Genetic differentiation (*F_ST_*) between cultivars of different geographic origins (N=271). Cultivars of unknown origin have been removed.

|  | Germany | UK | Australia | Belgium | Canada | Spain | France | Japan | New Zealand | Netherland | Russia | United-States | Switzerland | Tunisia | Ukraine |
| --- | --- | --- | --- | --- | --- | --- | --- | --- | --- | --- | --- | --- | --- | --- | --- |
| UK | 0.0089 |  |  |  |  |  |  |  |  |  |  |  |  |  |  |
| Australia | 0.0025 | -0.0142 |  |  |  |  |  |  |  |  |  |  |  |  |  |
| Belgium | 0.0379 | 0.0306* | 0.0095 |  |  |  |  |  |  |  |  |  |  |  |  |
| Canada | 0.0523 | 0.0269 | 0.0610 | 0.0865 |  |  |  |  |  |  |  |  |  |  |  |
| Spain | 0.0484 | 0.0001 | 0.0552 | 0.0230 | 0.1160 |  |  |  |  |  |  |  |  |  |  |
| France | 0.0260* | 0.0155* | -0.0007* | 0.0123 | 0.0153* | -0.0093 |  |  |  |  |  |  |  |  |  |
| Japan | -0.0008 | -0.0295 | 0.0137 | 0.0313 | 0.0156 | 0.0247 | 0.0020* |  |  |  |  |  |  |  |  |
| New Zealand | 0.0280 | 0.0168 | 0.0846 | 0.0618 | 0.1595 | 0.0893 | 0.0278* | 0.0463 |  |  |  |  |  |  |  |
| Netherland | 0.0116 | 0.0064 | 0.0258 | -0.0199 | 0.0430 | 0.0233 | 0.0071 | 0.0237 | -0.0080 |  |  |  |  |  |  |
| Russia | 0.0377* | 0.0369* | 0.0155 | 0.0318 | -0.0409 | -0.0011 | 0.0332* | -0.0359 | 0.0230 | 0.0354 |  |  |  |  |  |
| United States | 0.0168 | 0.0248* | 0.0198 | 0.0314* | 0.0744 | 0.0290* | 0.0299* | 0.0045 | -0.0062 | -0.0043 | 0.0369* |  |  |  |  |
| Switzerland | 0.0259 | -0.0089 | 0.0699 | 0.0750 | 0.5647 | 0.2087 | 0.0283 | 0.0235 | 0.2575 | 0.0489 | 0.0219 | -0.0177 |  |  |  |
| Tunisia | 0.0838 | 0.0575* | 0.0796 | 0.0901 | 0.2072 | 0.0969 | 0.0517* | 0.0695 | 0.1158 | 0.0782* | 0.0229 | 0.0294 | 0.1877* |  |  |
| Ukraine | 0.0726 | 0.0365* | 0.0502 | 0.1295* | 0.1183 | 0.1345 | 0.0629* | 0.0280 | 0.1334 | 0.1029* | 0.0262 | 0.0757* | 0.1323 | 0.1415 |  |
| Israel | 0.0267 | -0.0766 | 0.0631 | 0.0767 | 0.5122 | 0.1384 | -0.0221 | -0.0793 | 0.1539 | 0.0184 | -0.0019 | -0.0343 | 0.4605 | 0.1828 | 0.1293 |
| . * : P<0.05 | |  |  |  |  |  |  |  |  |  |  |  |  |  |  |
