# Supplementary material for: New Insight into the History of Domesticated Apple: Secondary Contribution of the European Wild Apple to the Genome of Cultivated Varieties
Source: PLoS Genet. 2012 May 10;8(5):e1002703. doi: 10.1371/journal.pgen.1002703 (PMC3349737; doi:10.1371/journal.pgen.1002703)
Supplement: Table S9 — Description of the Multiplex PCRs (MP01, MP02, MP03, MP04) used for microsatellite amplification. (DOC) [file pgen.1002703.s012.doc]

Table S9: Description of the Multiplex PCRs (MP01, MP02, MP03, MP04) used for microsatellite amplification.

| **MP01** |  |  |  | **MP02** |  |  |  | **MP03** |  |  |  | **MP04** | |  |  |
| --- | --- | --- | --- | --- | --- | --- | --- | --- | --- | --- | --- | --- | --- | --- | --- |
|  |  | Volume (µL) |  |  |  | Volume (µL) |  |  |  | Volume (µL) |  |  | |  | Volume (µL) |
| Mix qiagen |  | 7.50 |  | Mix qiagen |  | 7.5 |  | Mix qiagen |  | 7.5 |  | | Mix qiagen |  | 7.5 |
|  |  |  |  |  |  |  |  |  |  |  |  | |  |  |  |
| CH01h10 (10µM) | VIC | 0.15 |  | NZ05g08 (20µM) | HEX | 0.30 |  | Hi02c07 (10µM) | VIC | 0.15 |  | | CH04c07 (10µM) | VIC | 0.15 |
|  |  | 0.15 |  |  |  | 0.30 |  |  |  | 0.15 |  | |  |  | 0.15 |
| CH01h01 (10µM) | PET | 0.15 |  | CH05f06 (10µM) | PET | 0.30 |  | CH01f02 (20µM) | 6FAM | 0.30 |  | | GD12 (10µM) | PET | 0.15 |
|  |  | 0.15 |  |  |  | 0.30 |  |  |  | 0.30 |  | |  |  | 0.15 |
| CH01f03b (10µM) | 6-FAM | 0.15 |  | CH02d08 (10µM) | NED | 0.15 |  | CH02c11 (10µM) | NED | 0.15 |  | | CH03d07 (20µM) | 6FAM | 0.30 |
|  |  | 0.15 |  |  |  | 0.15 |  |  |  | 0.15 |  | |  |  | 0.30 |
| CH02c06 (20µM) | NED | 0.30 |  | CH04e05 (20µM) | 6-FAM | 0.30 |  |  |  |  |  | | CH02c09 (10µM) | NED | 0.15 |
|  |  | 0.30 |  |  |  | 0.30 |  |  |  |  |  | |  |  | 0.15 |
|  |  |  |  |  |  |  |  |  |  |  |  | |  |  |  |
| dWater |  | 4.00 |  | dWater |  | 3.40 |  | dWater |  | 4.30 |  | | dWater |  | 4 |
|  |  |  |  |  |  |  |  |  |  |  |  | |  |  |  |
| Total (without DNA) |  | 13.00 |  | Total (without DNA) |  | 13.00 |  | Total (without DNA) |  | 13.00 |  | |  |  |  |
| DNA |  | 2.00 |  | DNA |  | 2.00 |  | DNA |  | 2.00 |  | | Total (without DNA) |  | 13 |
| Total |  | 15.00 |  | Total |  | 15.00 |  | Total |  | 15 |  | | DNA |  | 2 |
|  |  |  |  |  |  |  |  |  |  |  |  | | Total |  | 15 |
