# Supplementary material for: New Insight into the History of Domesticated Apple: Secondary Contribution of the European Wild Apple to the Genome of Cultivated Varieties
Source: PLoS Genet. 2012 May 10;8(5):e1002703. doi: 10.1371/journal.pgen.1002703 (PMC3349737; doi:10.1371/journal.pgen.1002703)
Supplement: Text S1 — Method used for approximate Bayesian computations on alternative datasets/admixture times. (DOC) [file pgen.1002703.s013.doc]

**Text S1**

**Approximate Bayesian computations on alternative datasets/admixture times**

We conducted two additional sets of approximate Bayesian computations: (i) on a pruned dataset with misclassified wild individuals and individuals with a recent admixed ancestry removed, (ii) on the full dataset, but assuming that admixture between ancestral *M. domestica* and *M. sylvestris* was more recent (67 generations, 500 ybp) than in original analyses (200 generations, 1500 ybp). Analyses were conducted using the same prior sets than the main dataset (Table S3). In analyses on the pruned dataset, relative posterior probabilities of models *b* and *c* were not significantly different from each other (Table S4; model *b*: 0.5135, 95% confidence interval: 0.4778-0.5492; model *c*: 0.4857, 95% confidence interval: 0.4500-0.5214). However, since introgression between *M. domestica* and *M. orientalis* could not be estimated under model *b* (not shown), only parameter estimates for model *c* are reported in Table S5. In analyses assuming an alternative admixture time between *M. domestica* and *M. sylvestris*, the posterior probability of model *b* was only slightly lower than that of model *c* (Table S4), but, again, introgression between *M. domestica* and *M. orientalis* could not be estimated accurately under model *b* (model *b*: 0.5486, 95% confidence interval: 0.5089-0.5882; model *c*: 0.4154, 95% confidence interval: 0.3765-0.4543). Both analyses resulted in very minor changes to the point estimates for all parameters (Table S5).

**Model checking**

We assessed the goodness-of-fit of all model parameter posterior combinations. For each combination, 100 datasets were simulated using parameter values drawn posterior distributions. Summary statistics of the observed data were then ranked against the distributions obtained from simulated datasets (Cornuet et al. 2010). To avoid overestimating the quality of the fit by using the same statistics twice, model checking was based on test quantities summary statistics that have not been used in parameter inferences. Results are shown in Table S6. We found that none of the test quantities had significant tail-area probabilities under model *a*, *b*, *c*. Two test quantities (proportion of shared alleles between *M. domestica* and *M. sylvestris*, and between *M. orientalis* and *M. sieversii*) showed significant, or marginally significant, tail-area probabilities under model *d*. These results suggest that observed data are more plausible under the posterior predictive distributions generated under admixture models *a*, *b* and *c*, than under the posterior predictive distributions generated under model *d*.

**Confidence in model choice**

The performance of the method to discriminate among competing historical models was assessed by analyzing test datasets simulated with the same number of loci and individuals than in observed datasets (*i.e.*, pseudo-observed datasets). One hundred of such test data sets were simulated under each competing model, using parameter values drawn in the same prior distributions than those for original analyses. Relative posterior probabilities of competing models were evaluated for each pseudo-observed dataset, using the same methodology as described for the observed dataset. Confidence in model choice was then estimated using the proportion of cases a given scenario has not the highest posterior probability among competing scenarios when it is actually the true scenario (type I error) and the proportion of cases a given scenario has the highest posterior probability when it is actually not the true scenario (type II error).

For main analyses, results indicated a good power of our methodology to discriminate among the four competing models. For model *c*, the type I error rate amounted to 0.54, and the mean type II error rate was 0.05 (range: 0 – 14). Analyses on alternative datasets/parameter sets also indicated a good power to discriminate among competing models. For model *c* with the pruned dataset, the type I error rate amounted to 0.57 and the mean type II error rate was 0.08 (range: 2-19). For model *c* using an alternative admixture time, type I error rate was 0.47, and the mean type II error was 0.067 (range: 0 – 18).
